# Development of Covalent Chitosan-Polyethylenimine Derivatives as Gene Delivery Vehicle: Synthesis, Characterization, and Evaluation

**DOI:** 10.3390/ijms22083828

**Published:** 2021-04-07

**Authors:** Laura Nicolle, Jens Casper, Melanie Willimann, Céline M. A. Journot, Pascal Detampel, Tomaž Einfalt, Hiu Man Grisch-Chan, Beat Thöny, Sandrine Gerber-Lemaire, Jörg Huwyler

**Affiliations:** 1 Group for Functionalized Biomaterials, Institute of Chemical Sciences and Engineering Ecole Polytechnique Fédérale de Lausanne, EPFL SB ISIC SCI-SB-SG, Station 6, CH-1015 Lausanne, Switzerland; laura.nicolle@epfl.ch (L.N.); celine.journot@epfl.ch (C.M.A.J.); 2 Division of Pharmaceutical Technology, Department of Pharmaceutical Sciences, University of Basel, Klingelbergstrasse 50/70, CH-4056 Basel, Switzerland; jens.casper@unibas.ch (J.C.); pascal.detampel@unibas.ch (P.D.); tomaz.einfalt@unibas.ch (T.E.); 3 Division of Metabolism and Children’s Research Center, University Children’s Hospital Zürich, CH-8032 Zürich, Switzerland; Melanie.Willimann@kispi.uzh.ch (M.W.); HiuMan.Grisch@kispi.uzh.ch (H.M.G.-C.); Beat.Thoeny@kispi.uzh.ch (B.T.)

**Keywords:** chitosan, polyethylenimine copolymer, DNA condensation, gene delivery vector

## Abstract

There is an increasing interest in cationic polymers as important constituents of non-viral gene delivery vectors. In the present study, we developed a versatile synthetic route for the production of covalent polymeric conjugates consisting of water-soluble depolymerized chitosan (dCS; M_W_ 6–9 kDa) and low molecular weight polyethylenimine (PEI; 2.5 kDa linear, 1.8 kDa branched). dCS-PEI derivatives were evaluated based on their physicochemical properties, including purity, covalent bonding, solubility in aqueous media, ability for DNA condensation, and colloidal stability of the resulting polyplexes. They were complexed with non-integrating DNA vectors coding for reporter genes by simple admixing and assessed in vitro using liver-derived HuH-7 cells for their transfection efficiency and cytotoxicity. Using a rational screening cascade, a lead compound was selected (dCS-Suc-LPEI-14) displaying the best balance of biocompatibility, cytotoxicity, and transfection efficiency. Scale-up and in vivo evaluation in wild-type mice allowed for a direct comparison with a commercially available non-viral delivery vector (in vivo-jetPEI). Hepatic expression of the reporter gene luciferase resulted in liver-specific bioluminescence, upon intrabiliary infusion of the chitosan-based polyplexes, which exceeded the signal of the in vivo jetPEI reference formulation by a factor of 10. We conclude that the novel chitosan-derivative dCS-Suc-LPEI-14 shows promise and potential as an efficient polymeric conjugate for non-viral in vivo gene therapy.

## 1. Introduction

Gene therapy has emerged in the last decades as a powerful strategy for the treatment of acquired and inherited disorders. Various diseases could benefit from it, including cancer and viral infections, cardiovascular diseases, and inherited Mendelian disorders [1,2]. This approach relies on the modification of gene expression by gene replacement, i.e., transfer of exogenous nucleic acids as a therapeutic gene copy, or precise genome editing [3]. Due to their polyanionic nature and their sensitivity to endonucleases, nucleic acids should preferably be delivered to target cells by means of nanocarriers, also called delivery vectors. These delivery vectors are mainly subdivided in two categories: viral and non-viral [4]. Viral delivery vectors display high transfection efficiency in vivo [5,6] but their inherent immunogenicity and/or cytotoxicity, and potential genome integration, remain major drawbacks as they may trigger severe adverse immune responses [4]. Non-viral vectors have less limitations for condensation of genetic material as compared to viral delivery vectors [7]. Consequently, the interest in synthetic non-viral vectors for gene delivery as a potentially safer alternative has steadily increased over the past years.

Non-viral vectors often contain cationic polymers or lipids. Upon condensation with polyanionic nucleic acids, they form polyplexes or lipoplexes, respectively [8,9]. Cationic polymers like polyethylenimine (PEI) can efficiently condense nucleic acids into nanometer-sized structures and are therefore often used for gene delivery [10,11,12]. Two chemical structures of PEI exist, namely branched (BPEI) and linear (LPEI) PEI. Whilst BPEI contains various primary, secondary, and tertiary amines, LPEI displays only secondary amines [11,13]. PEI is known for its high cationic charge density [14] enabling efficient condensation of nucleic acids via electrostatic interactions [10,15,16,17]. Most importantly, the high availability of amine groups of PEI displays a large buffering potential at physiological pH [14]. Consequently, PEI is believed to act as a “proton sponge”, favoring endosomal escape of the polyplexes [18]. Although still under debate [13,19,20], the “proton sponge” effect remains the main hypothesis to explain the high cytosolic availability and the enhancement of transfection efficiency of PEI-based systems. Therefore, branched PEI structures and high molecular weight derivatives were introduced to increase the charge density [21,22,23,24].

Unfortunately, high transfection efficiency of cationic polymers correlates with an increased cytotoxicity. PEI-mediated toxicity is attributed to its high charge density and poor biodegradability [23]. The introduction of shielding polymers, such as polyethylene glycol (PEG), is an approach to reduce cytotoxicity of PEI-based systems [25]. However, shielding of positive charges and overall stabilization through covalent crosslinking exerts the positive effects at the expense of reduced transfection efficiency [26,27]. It is therefore a challenging task to optimize the structure of cationic polymers to find the right balance between charge density/efficacy, cytotoxicity, and in vivo tolerability [24,28,29,30].

PEI can be combined with chitosan (CS), a cationic polysaccharide obtained by partial deacetylation of chitin [31,32]. CS is composed of repeating β-(1,4)-2-amino-D-glucose and β-(1,4)-2-acetamido-D-glucose units that are linked by 1,4-β-glycosidic bonds. For the past decades, CS has been largely investigated for various medical and industrial applications [33,34,35,36,37] including drug delivery [38,39]. It is known for its biodegradability [40,41], biocompatibility [42], and ability to condense DNA [4,41,43]. However, the low charge density of CS leads to poor transfection efficiency of CS/DNA systems [4]. Another commonly encountered issue is the poor solubility of CS in organic solvents. These limitations can be overcome by conjugating small functional groups or cationic polymers [4,42,44,45,46,47] to the amino and hydroxyl groups of glucosamine units.

**Figure 1:**
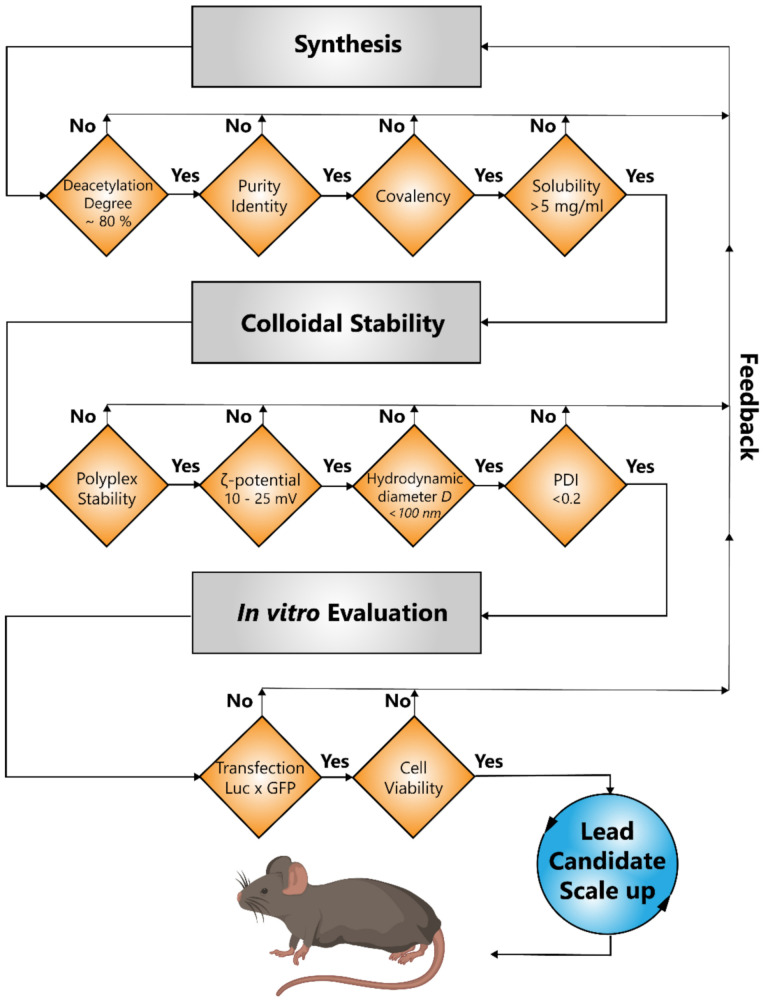
Screening cascade of depolymerized chitosan (dCS)–PEI–based delivery vectors. Identification of a lead candidate was guided by defined chemical properties of the polymer, the indicated physicochemical characteristics of the polyplexes to be met, and biological performance in vitro (i.e., transfection efficiency and cytotoxicity) using the hepatic HuH–7 cell line. Following synthesis, the resulting polymer was chemically identified by ^1^H NMR and tested for covalent bonding by 2D–diffusion ordered spectroscopy (DOSY) NMR. Only compounds with a solubility of >5 mg/mL were selected for further characterization. Colloidal stability was assessed after DNA complexation. In vitro performance was evaluated by using two reporter gene vectors (n.CAGLuc2 and n.CAG.GFP1). The lead candidate is characterized by favorable safety and transfection profiles in vitro allowing for in vivo evaluation of transfection efficiency.

The aim of the present study was to design, synthesize, and evaluate novel cationic polymers by using CS as a biocompatible, versatile, and biodegradable backbone covalently modified with low molecular weight PEI chains. As building blocks, depolymerized CS (dCS; M_W_ 6–9) and low molecular weight PEI chains (2.5 kDa linear, 1.8 kDa branched PEI) were used. Based on our recent work on CS depolymerization [48], we used water-soluble dCS chains for controlled covalent conjugation with PEI chains via a succinyl (Suc) spacer. A screening cascade (Figure 1) was implemented to guide medicinal chemistry efforts by defining physicochemical parameters required for the covalent polymer candidates. Subsequently, covalent polymer candidates were screened in cell culture using the HuH-7 hepatic cell line to optimize transfection efficiency and to minimize cytotoxicity. A lead candidate was identified using this screening approach, further scaled up, and confirmed in mice to have a high transfection efficiency of the liver.

## 2. Results

### 2.1. Synthesis and Physicochemical Properties of Covalent dCS-PEI Derivatives

**Figure 2:**
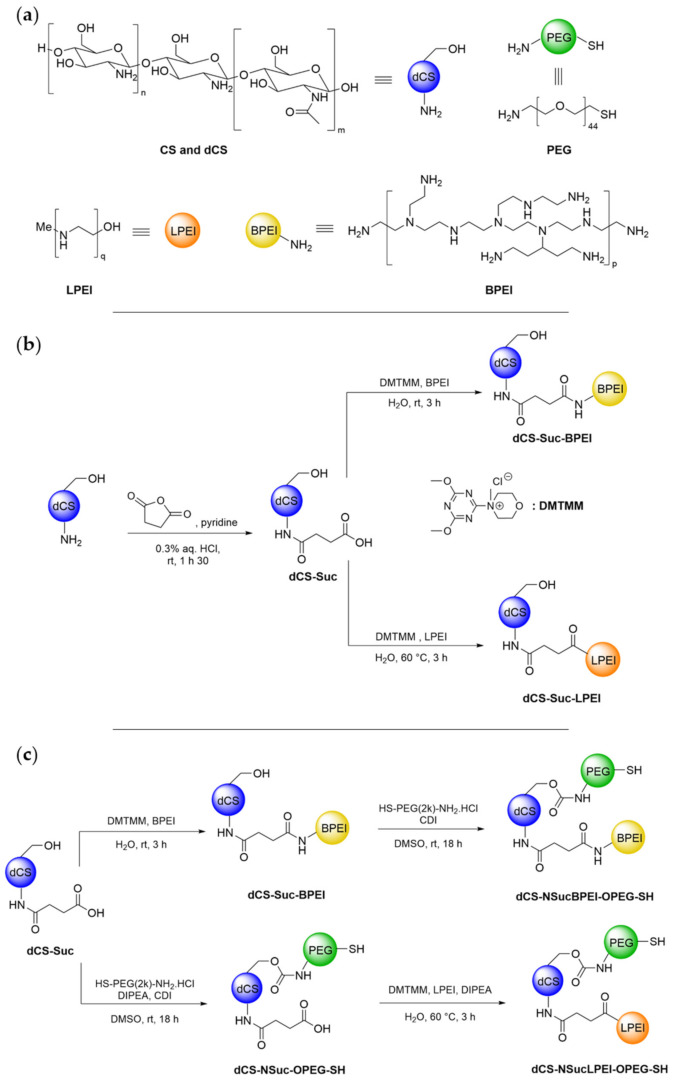
Synthesis of covalent dCS-PEI conjugates. (**a**) Structures of chitosan (CS, DD ≈ 80%), depolymerized chitosan (dCS, DD ≈ 80%), polyethylene glycol (PEG, 2 kDa), and branched (BPEI, 1.8 kDa) and linear (LPEI, 2.5 kDa) polyethylenimine. (**b**) Synthesis of dCS-Suc-BPEI and dCS-Suc-LPEI using a succinyl linker (Suc) and DMTMM as water-soluble coupling agent. (**c**) Synthetic pathways for sequential grafting of BPEI, LPEI, and H_2_N-PEG-SH on dCS-Suc. NSuc: Suc linked to amine groups. OPEG: PEG linked to hydroxyl groups. For detailed representation of the PEI conjugation sites to dCS-Suc, see supporting information, Figure S1.

A synthesis strategy was developed to conjugate dCS as biodegradable backbone with low molecular weight PEI chains to generate efficient and tolerable delivery vectors (Figure 2a). Based on the literature about CS functionalization [49,50,51], the reactivity of a commercial CS batch (mixture of three CS distributions with M_W_ 479, 88, and 45 kDa; determined by gel permeation chromatography (GPC) analysis) was investigated by activating its primary amines and then condensing BPEI on it through covalent urea or amide linkages. These attempts were not met with success and pointed out the limited solubility of commercial CS in organic solvents (<5 mg/mL), thus hampering the development of standardized functionalization procedures. Recently, our group disclosed a microwave-assisted methodology for the fast and controlled depolymerization of CS under acidic conditions, allowing the selection of the polymer’s average molecular weight via isolation of the soluble fraction at different pH values [48]. Following this methodology, depolymerized chitosan (dCS) batches with low polydispersity index and average molecular weight (M_W_) ranging from 6 to 9 kDa were produced (cf. supporting information S-6 for NMR and GPC data), allowing reliable comparison of the copolymers after functionalization with PEI. Conjugation to BPEI was initially attempted via direct activation of dCS primary amines in the presence of 1,1′-carbonyldiimidazole (CDI) or N,N’-disuccinimidyl carbonate (DSC), followed by condensation with the BPEI units and formation of urea as covalent linkage (Table S1, Scheme S1). For both products, only low grafting degrees (GD) and poor solubility were observed. Therefore, a succinyl (Suc) spacer was introduced for further functionalization via carboxylic acid functionality (Figure 2b).

The resulting intermediate dCS-Suc (GD_Suc_ = 40–60%) displayed solubility greater than 10 mg/mL in both aqueous solution and DMSO, enlarging the functionalization possibilities. For both BPEI and LPEI, the optimization of coupling agents, reaction time, and temperature finally led to pure dCS-Suc-BPEI and dCS-Suc-LPEI with 10–15% GD of the PEI chains (Figure 2b, Table S1 and Table 1). A higher GD_BPEI_ (67%) was achieved by increasing the proportion of BPEI and the coupling agent (**dCS-Suc-BPEI-67**, Table 1). Importantly, the homogeneity of dCS-Suc and the final conjugates was improved by centrifugation of the purified derivatives (solution obtained after dialysis), followed by collection and lyophilization of the supernatant. This led to water-soluble final derivatives. Analysis of the precipitates from the centrifugation step revealed also the presence of **dCS-Suc-PEI** copolymers but with dCS/PEI ratios differing from the values measured in the supernatant. Additionally, those derivatives did not lead to homogeneous solutions in pure distilled water, compromising further biological assessment. These findings pointed out the need to only collect the supernatant obtained after centrifugation for all synthetic intermediates and final derivatives. The detailed characteristics of the polymeric conjugates, which were screened for gene delivery applications, are listed in Table 1.

Anticipating that dCS-Suc-PEI copolymers might have cytotoxic properties due to their residual positive charge after DNA condensation, we considered additional derivatization of the primary alcohols of dCS with linear PEG derivatives (Figure 2c). The end thiol functionality of the PEG chain was selected for further potential stabilization of the polyplexes by chemical cross-linking resulting from disulfide bridge formation. Interestingly, the optimal sequence for this dual functionalization pathway was reversed depending on the PEI structure (linear or branched; Table S2, Scheme S2). In both cases, activation of dCS primary alcohols with CDI, followed by in situ trapping of the imidazolide intermediate with H_2_N-PEG-SH, resulted in covalent conjugation of PEG via a carbamate linkage, with a GD ranging from 10 to 36%.

Following their syntheses, the polymeric conjugates were characterized in detail as shown in Table 1, along with the starting polymers in order to compare their chemical composition and solubility. The GD of the polymeric conjugates was calculated for each component and the weight average of Suc, PEI, and PEG units were compared to the total molecular weight (w%) of the derivatives. The solubility in neutral to slightly acidic aqueous solutions was a crucial parameter to qualify the conjugates for further evaluation as delivery vectors. Therefore, a minimum solubility threshold of 5 mg/mL was set to achieve efficient DNA condensation. Apart from **dCS-Suc-BPEI-13** (BPEI-functionalized dCS with a grafting degree of 13%; Table 1), the solubility value of all polymeric conjugates exceeded 10 mg/mL. Interestingly, dCS and LPEI displayed limited solubility in aqueous solution (3 and 5 mg/mL, respectively), whereas their covalent conjugation into **dCS-Suc-LPEI-11a** and **dCS-Suc-LPEI-11b** resulted in a considerable increase of solubility. Apart from **dCS-Suc-LPEI-11a**, each copolymer detailed in Table 1 was obtained after lyophilization of the supernatant fraction collected from the post-dialysis centrifugation (cf. materials and methods for detailed procedures).

### 2.2. NMR Analyses of Covalent dCS-PEI Derivatives

**Figure 3:**
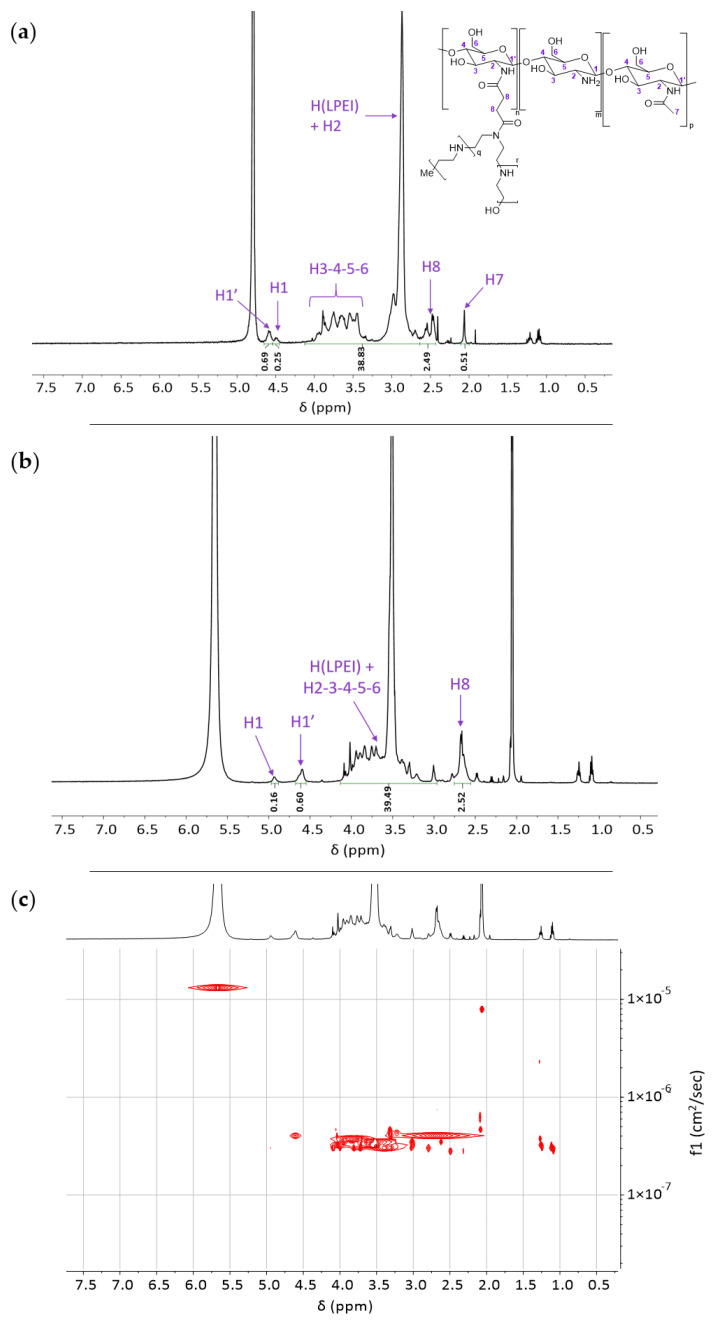
NMR characterization of **dCS-Suc-LPEI-14**. (**a**) ^1^H NMR spectrum in D_2_O and (**b**) in D_2_O/acetic acid-d^4^. (**c**) Determination of the covalent bonding of **dCS-Suc-LPEI-14** by 2D-DOSY NMR in D_2_O/acetic acid-d^4^. For details on analyses and assignments, cf. Supporting information S-11.

The polymeric intermediates and final conjugates were characterized by ^1^H nuclear magnetic resonance (NMR) and 2D-diffusion ordered spectroscopy (2D-DOSY NMR) to confirm the composition, GD, and covalent functionalization for all synthesized derivatives. A representative example of the NMR characterization sequence is given for **dCS-Suc-LPEI-14** (Figure 3). Due to overlapping signals from the dCS backbone and LPEI units, the ^1^H NMR spectra were recorded in D_2_O (Figure 3a) and D_2_O/acetic acid-d^4^ (Figure 3b). The characteristic signal for LPEI -CH_2_ stands at 2.88 ppm (Figure 3a) and 3.52 ppm (Figure 3b). The determination of the GD_LPEI_ was based on the signal of the succinyl spacer (Figure 3b, H_8_), which was used as a reference for the integration of the massif of interest (4.13–2.97 ppm) reporting for LPEI -CH_2_ and glucosamine H_2_ to H_6_. Setting the integration of H_8_ at 2.52 ppm, GD_LPEI_ was calculated with the following equation:

$$GD_{LPEI}=\;\frac{H_{total}-H_{glucosamine}}{H_{LPEI}}\;\times \;100$$

where *H_total_* is the integrated signal 4.13–2.97 ppm, *H_glucosamine_* represents the sum of H_2_, H_3_, H_4_, H_5_, H_6_, and *H_LPEI_* is the total number of H in one LPEI chain (2.5 kDa) (cf. supporting information S-11 for detailed calculations). In the present case, a *GD_LPEI_* of 14% was calculated. Overall, the alignment of the diffusion constants for all signals of the molecular structures was confirmed for every polymeric conjugate (cf. supporting information S-6 to S-25 for NMR characterization of all synthetic derivatives).

### 2.3. In Vitro Performance of DNA Complexed Covalent dCS-PEI Derivatives

For in vitro assessment, the dCS-PEI derivatives and the polymers used as starting materials (category “Starting polymer” in Table 2) were included in the polyplex-formation analyses. The primary indicator of DNA condensation is evaluated by the amount of polymer required to bind DNA and shield it from external forces. The starting materials **dCS** and **LPEI** were observed to form stable complexes at c/p 32 (weight ratio of polymer (c) to nucleic acid (p); c/p ratio) and BPEI at c/p 16 (Table 2). In contrast, dCS-Suc-BPEI and dCS-Suc-LPEI derivatives reached stable polyplexes with DNA at considerably lower c/p ratios. Whereas dCS-Suc-BPEI derivatives require the lowest amount of polymer to generate stable polyplexes with values at c/p 0.5 (**dCS-Suc-BPEI-13**, **dCS-Suc-BPEI-67**) and c/p 1 (**dCS-Suc-BPEI-11**). In comparison, the dCS-Suc-LPEI derivatives formed stable polyplexes at c/p 1 (**dCS-Suc-LPEI-11a**) and c/p 2 (**dCS-Suc-LPEI-11b**). Furthermore, additional conjugation of PEG to the linear and branched dCS-Suc-PEI system did not alter the polyplex stability (**dCS-NSucLPEI-OPEG-SH** and **dCS-NSucBPEI-OPEG-SH**). The polymeric systems synthesized within this study improved the DNA condensation ability by 8–64-fold compared to starting polymers **dCS**, **LPEI**, and **BPEI.**

To ensure efficient cellular uptake and cellular trafficking of polyplexes, a moderately positive surface potential, small size, and low polydispersity are required [52,53,54,55]. The optima for these colloidal properties do not necessarily coincide with the amount of dCS-PEI derivatives required to form stable polyplexes. At the abovementioned c/p ratios, ζ-potential of polyplexes can be negative or close to neutral, leading to aggregate formation. Therefore, the optimal c/p ratio resulted in small-sized, monodispersed (narrow particle size distribution) particles with slightly positive ζ-potential. Colloidally stable polyplexes were defined by a ζ-potential between 10 and 25 mV, a hydrodynamic diameter under 100 nm, and a polydispersity index (PDI) below 0.2. The starting polymers had similar values for colloidal stability at c/p 16 (**dCS**, **LPEI**) and 32 (**BPEI**) as previously observed using agarose gel electrophoresis. The dCS-Suc-LPEI derivatives required the same amounts of polymer to form colloidally stable particles. However, dCS-Suc-BPEI polyplexes required two- (**dCS-Suc-BPEI-11** and **dCS-Suc-BPEI-67**) to eight-fold (**dCS-Suc-BPEI-13**) higher amounts. Interestingly, additional conjugation of PEG only marginally altered the colloidal stability of the polyplexes. At the corresponding c/p ratio of the starting polymers, only LPEI failed the requirements displaying a ζ-potential of 28 mV. Interestingly, all LPEI and BPEI derivative-based polyplexes demonstrated a surface charge within the requirements. Some polyplexes displayed a lower ζ-potential compared to the corresponding starting polymers (e.g., 16 mV, **dCS-Suc-BPEI-13**), which was further reduced by the conjugation of PEG (13 mV, **dCS-NSucBPEI-OPEG-SH**). Almost all polyplexes displayed a hydrodynamic diameter under 100 nm including polyplexes formed by starting polymers (Table 2). The group of dCS-Suc-BPEI-based polyplexes decreased marginally in size compared to BPEI-based polyplexes, whereas dCS-Suc-LPEI derivative-based polyplexes were bigger compared to LPEI-based polyplexes. As expected, the additional conjugation of PEG onto dCS-Suc-BPEI led to bigger polyplexes (121 nm), whereas dCS-NSucLPEI-OPEG-SH-based polyplexes displayed a similar size compared to dCS-Suc-LPEI-based polyplexes. Polyplexes based on starting polymers showed a PDI < 0.2. In particular, **dCS-Suc-LPEI-11b** and **dCS-NSucLPEI-OPEG-SH** showed a homogeneous particle size distribution (PDI < 0.2). In contrast, dCS and most of the dCS-Suc-LPEI-based and dCS-Suc-BPEI-based polyplexes displayed values > 0.2.

The polyplexes were further evaluated by the DNA exclusion assay to determine whether the complexation itself provides protection from external factors. A c/p of 32 for dCS/DNA showed complete shielding of the DNA. Interestingly, polyplexes of LPEI c/p 32 and BPEI c/p 16 showed residual accessibility of DNA resulting in 6% ± 1% and 5% ± 3% of unshielded DNA, respectively. However, the other derivative groups with and without conjugated PEG displayed complete DNA encapsulation (Table S3).

We next assessed the transfection efficiency of the delivery vectors using luciferase and GFP encoding nanovector-DNAs using the same polymer/DNA ratio determined as optimal regarding its colloidal stability. To ensure a rapid screening, only c/p ratios of polyplexes were tested, which showed optimal colloidal stability. In order to compare luciferase and GFP transfections levels, data were normalized to dCS c/p 32 polyplexes (baseline value, i.e., 1). The transfection efficiency of the starting polymers LPEI and BPEI improved by over 2000- and 3000-fold compared to dCS, respectively (Table 2). Whilst the transfection efficiency was almost the same for **dCS-Suc-BPEI-67**, **dCS-Suc-BPEI-11**, and **dCS-Suc-BPEI-13**-based polyplexes were inferior compared to BPEI-based polyplexes. With regard to dCS-Suc-LPEI derivative-based polyplexes, both derivatives increased the transfection efficiency in comparison to their starting polymers **dCS** and **LPEI**. Significant improvement of transfection efficiency was measured for **dCS-Suc-LPEI-11b** resulting in an over 70,000-fold increase compared to **dCS** and 34-fold to **LPEI**, respectively. Unexpectedly, the derivatives conjugated with PEG resulted in a complete loss of transfection.

Lastly, the effect of the polyplexes on cell viability was assessed using the hepatic cell line HuH-7. In the cell viability assay on the one hand, LPEI and BPEI-based polyplexes yielded only 25% and 43% viable cells, respectively. On the other hand, the biocompatible and biodegradable dCS polyplexes showed no detrimental effect on cell viability. A significant increase in cell viability resulted from the covalent conjugation of dCS to PEI (min 61% for **dCS-Suc-BPEI-67**, max 101% for **dCS-Suc-LPEI-11b**). Additional conjugation of PEG had beneficial effects on BPEI-based polyplexes but showed no clear trend for LPEI-based polyplexes.

Considering the different characteristics of polyplexes for colloidal stability, in vitro transfection and tolerability, **dCS-Suc-LPEI-11b** showed the most favorable properties and was therefore selected as the lead candidate. At that stage, the functionalization of dCS-Suc-PEI conjugates with PEG was shown to require further optimization, since the transfection efficiency was severely reduced and the shielding did not induce beneficial effects on cell viability.

### 2.4. Selection of a Lead Compound

Based on in vitro results (cf. Section 2.3) and following the screening procedure detailed in Figure 1, **dCS-Suc-LPEI-11b** was identified as a lead compound. This lead candidate did meet all criteria defined by the screening cascade including chemical properties of the copolymer, physicochemical characteristics of the polyplex referred to as colloidal stability (i.e., ζ-potential, hydrodynamic diameter *D*, and PDI), and biological performance in vitro. The stability assay (i.e., an assessment of the extent of nucleic acid complexation by agarose gel electrophoresis) allowed us to determine the amount of copolymer required to successfully condense DNA. The lead candidate was then synthesized on a larger scale (≈ 900 mg) to allow for mechanistic studies and in vivo investigations. From here onward, the scaled-up batch of **dCS-Suc-LPEI-11b** will be referred to as **dCS-Suc-LPEI-14** (LPEI-functionalized dCS with a grafting degree of 14%, Table 1). Furthermore, we assessed the reproducibility of the whole procedure by conducting each reaction on several parallel batches, which were combined after validating their characteristics: in total, four batches of 1 g were produced for the preparation of **dCS-Suc** (GD_Suc_ 63%, yield 67%) and seven batches of 0.2 g for **dCS-Suc-LPEI-14** (GD_LPEI_ 14%, yield 35%; cf. Table S4).

### 2.5. In Vitro Gene Expression and Cytotoxicity Evaluation of the Lead Candidate

**Figure 4:**
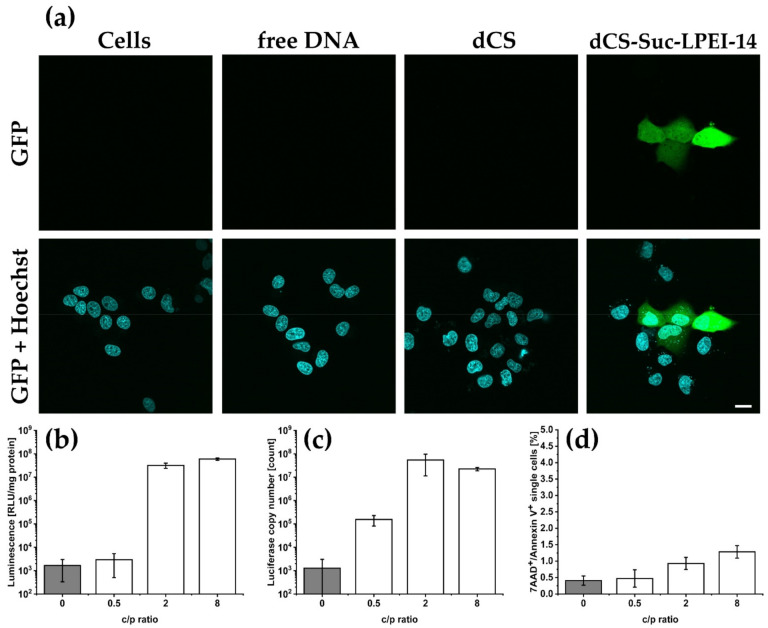
In vitro gene expression and toxicity pattern of the lead candidate. Experiments were conducted using two reporter gene nanovector-DNA (n.CAGLuc2 and n.CAG.GFP1). (**a**) Left Panel: Untreated control cells. Nuclei were stained with Hoechst 33,342 (Blue). Middle Panels: Expression of GFP in HuH-7 cells 48 h after transfection with free DNA and dCS. Right panel: Expression of GFP in HuH-7 cells 48 h after transfection with lead candidate **dCS-Suc-LPEI-14**. Scale bar: 20 μm. (**b**–**d**) In vitro evaluation of HuH-7 cells at c/p ratios 0 (free DNA), 0.5, 2, and 8: (**b**) quantitative assessment of luciferase expression as a function of c/p ratios. Gene expression of luciferase is based on luciferin conversion. (**c**) Delivery of luciferase reporter genes as determined by quantitative polymerase-chain reaction (qPCR). (**d**) Evaluation of late apoptotic and necrotic cells 48 h after incubation with **dCS-Suc-LPEI-14**. Values are means ± SD, *n* ≥ 3.

The lead candidate **dCS-Suc-LPEI-14** was further evaluated. Different c/p ratios were investigated with focus on transfection efficiency and potential cytotoxic effects. Used methods included confocal microscopy, luciferin conversion, quantitative polymerase-chain reaction (qPCR), and flow cytometry to distinguish between apoptosis and necrosis (Figure 4a–d). Transfection using **dCS-Suc-LPEI-14**-based polyplexes led to an isolated, but strong expression of the fluorescent reporter GFP at c/p 2 as compared to control (i.e., untreated cells), transfection with naked DNA, or **dCS** c/p 32 (Figure 4a). We observed non-homogenous distribution of GFP signals, which corresponded with the findings from flow cytometry experiments (see Figure S2) and with previous observations with **dCS-Suc-LPEI-11b**. Depending on the c/p ratio, up to 34% of the cells were transfected. The polyplexes were further evaluated with luciferase-encoding nanovector-DNA as a preliminary test for further in vivo experiments. The luciferase transfection using **dCS-Suc-LPEI-14** resulted in 3.2 × 10^7^ RLU/mg protein for c/p 2 and only marginally increased with higher amounts of polymer (c/p 8; Figure 4b). A similar observation was made when luciferase copy numbers were determined by qPCR (Figure 4c). The luciferase copy numbers increased by almost three orders of magnitude when transfected with polyplexes of c/p 0.5 to c/p 2 and plateaued at c/p 2. To determine the number of apoptotic and necrotic cells, 7-AAD (7-aminoactinomycin D) and Annexin V double-labeling was conducted and analyzed by flow cytometry (Figure 4d). Compared to cells treated with DNA only, **dCS-Suc-LPEI-14** showed marginal elevation of 7-AAD and Annexin V positive cells. The in-depth characterization of the scaled-up lead candidate **dCS-Suc-LPEI-14** confirmed the results from the screening experiments and qualified this derivative for testing in wild-type mice.

### 2.6. In Vivo Assessment of the Lead Candidate

**Figure 5:**
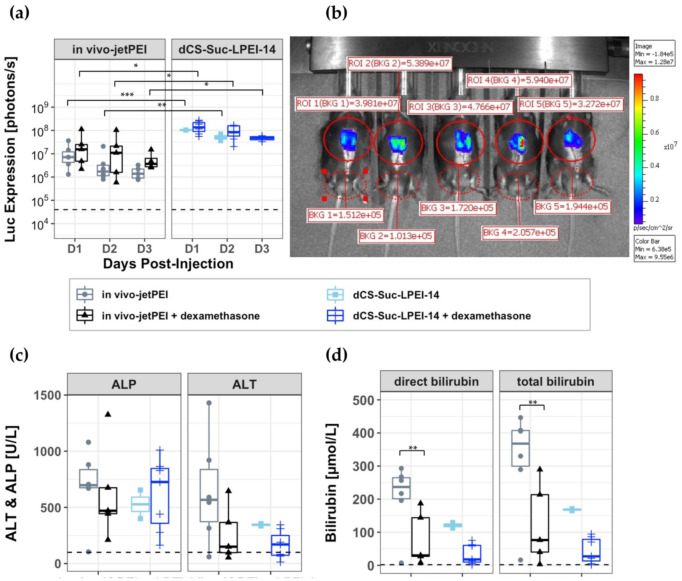
In vivo assessment of **dCS-Suc-LPEI-14** upon retrograde intrabiliary infusion into wild-type mice. (**a**) Luciferase expression levels up to three days post retrograde intrabiliary infusion of 1 µg nanovector-DNA encapsulated with a c/p ratio of 2 (**dCS-Suc-LPEI-14**) and 1.1 (in vivo-jetPEI). Animals were pretreated without (*n* = 6) or with dexamethasone (*n* = 5) for in vivo-jetPEI, and without (*n* = 2) or with dexamethasone (*n* = 5) for **dCS-Suc-LPEI-14**. Note that mice injected without dexamethasone showed signs of jaundice and severe cases had to be euthanized (and were therefore not screened for IVIS). (**b**) Detection of liver-associated bioluminescence in mice three days post intrabiliary infusion (1 µg DNA/**dCS-Suc-LPEI-14** + dexamethasone). (**c**,**d**) Serum markers for hepatotoxicity, including alkaline phosphatase (ALP), alanine transaminase (ALT), total bilirubin, and direct bilirubin. Statistical differences by Student’s two tailed *t*-test: * = *p* < 0.05, ** = *p* < 0.01, *** = *p* < 0.001. Dashed line: background = untreated mice. Values are means ± SD, *n* ≥ 3.

The in vivo transfection efficacy of **dCS-Suc-LPEI-14** complexed with luciferase encoding nanovector-DNA was assessed in wild-type mice. Complexes of **dCS-Suc-LPEI-14** at a c/p ratio of 2.0 were formed using 1 μg up to 50 µg of nanovector-DNA (n.P3Luc1) expressing the luciferase from the liver-specific promoter P3. For comparison, we used the commercially available non-viral delivery vector in vivo-jetPEI [56] and encapsulated 1 µg n.P3Luc1 at c/p 1.1 according to the manufacturer’s protocol. The polyplexes were administered intrabiliary as these derivatives are not designed to target the liver upon systemic injection. The systemic administration was investigated in a pilot experiment showing that intravenous injection of the **dCS-Suc-LPEI-14**-based polyplexes did not result in any luciferase expression up to day three (10 µg and 50 µg nanovector-DNA injected intravenous, data not shown). Therefore, the polyplexes containing 1 µg nanovector-DNA (and 5 µg and 10 µg nanovector-DNA, data not shown) were administered by retrograde infusion through the gallbladder for liver cell transfection. In vivo bioluminescence imaging (IVIS) was followed at day one, two, and three post infusion (Figure 5a,b). Mice injected with in vivo-jetPEI showed an average luciferase expression of 6.4 × 10^6^ photons/s over a period of three days, whereas mice injected with **dCS-Suc-LPEI-14** polyplexes showed an enhanced luciferase signal of 7.9 × 10^7^ photons/s. Thus, the use of **dCS-Suc-LPEI-14** polyplexes exceeded the luciferase expression by more than ten-fold compared to in vivo-jetPEI (Figure 5a). Furthermore, luciferase expression was confined to be specific to the liver based on in vivo imaging system (IVIS) (Figure 5b). Subsequently the same polyplexes were tested upon pre-administration of the anti-inflammatory drug dexamethasone [57] in an effort to suppress liver toxicity and jaundice in mice. The dexamethasone treated mice exhibited a slight increase of luciferase expression compared to non-treated ones, and again a higher expression for the **dCS-Suc-LPEI-14** polyplexes compared to the in vivo-jetPEI control was observed, i.e., 1.1 × 10^8^ p/s versus 2.3 × 10^7^ p/s, respectively. Levels of standard liver markers were determined in the serum of mice upon euthanization at day 3. Bilirubin and ALT levels could be reduced by dexamethasone pretreatment while ALP levels were not significantly affected.

## 3. Discussion

In the present study, CS was used as a backbone for the design of biodegradable and biocompatible PEI derivatives. Functionalization was achieved by covalent modification of amino and hydroxyl groups of CS units. To overcome the poor water solubility of CS, we used a small molecular weight depolymerized CS recently introduced [48]. Furthermore, the depolymerization of CS led to a homogenous polymer size distribution of the resulting low molecular weight dCS preparations. This was shown to be essential for reproducible functionalization and further processing since copolymers isolated from conjugation with the commercial CS contained agglomerates of molecules with highly variable molecular weights. Using water-soluble dCS as a starting polymer allowed us to develop synthetic pathways for the sequential coupling of PEI and PEG chains. The introduction of a succinyl spacer was a prerequisite to use an amide bond formation coupling agent like DMTMM (4-(4,6-dimethoxy-1,3,5-triazin-2-yl)-4-methyl-morpholinium chloride). Due to the high water-solubility of DMTMM, BPEI and LPEI covalent grafting to dCS-Suc could be easily carried out in water with great reproducibility (Figure 2 and Table S4). This synthetic strategy is in contrast to usual ones, where the EDC/NHS coupling agent system is more frequently used to form amide bonds [58]. However, in our case DMTMM led to better and higher grafting degree than EDC/NHS (Table S1). In order to improve the homogeneity and purity of the final conjugates, a centrifugation step had to be introduced after purification by dialysis to remove precipitated polymer aggregates. The impact of this additional treatment on the properties of the copolymers was assessed by comparing **dCS-Suc-LPEI-11a** (not separated by centrifugation) and **dCS-Suc-LPEI-11b** (separated by centrifugation) as shown by a decrease in PDI by 35% and the significant differences with respect to their in vitro performance (Table 2).

^1^H and 2D-DOSY NMR spectroscopies played a central role in the characterization of dCS-based conjugates, allowing not only for the determination of their molecular structure (i.e., composition and grafting degree) but also for the confirmation of the covalent conjugation of all polymeric components (Figure 2). In the present study, we first characterized the different systems by ^1^H NMR analysis in up to two solvents (pure D_2_O and D_2_O/acetic acid-d^4^), and then ensured their covalent bonding by subjecting them to 2D-DOSY NMR experiments. Confirmation of covalent bonding is of high importance since the assembly of polymers through non-covalent interactions can lead to stable structures resistant to purification by dialysis. Several CS-PEI based systems were already developed for gene delivery in the past 20 years, but their characterization was focused on GPC, FTIR, and ^1^H NMR, lacking interpretation for covalent bonding [59,60,61]. In particular, ^1^H NMR spectra of polymer aggregates may only slightly differ from their covalent analogues and GPC analysis is insufficient since branched polymers cannot be identified by this technique. We believe that the NMR sequence introduced here is crucial to distinguish between covalent and non-covalent assemblies. This methodology can be applied to a large variety of multicomponent copolymers, giving important insight into their molecular structure in relation with their stability and degradation profile in the physiological environment.

As a result of the synthetic chemical effort (Figure 2), six promising candidates could be selected for screening in cell culture models (Table 1 and Table 2). All candidates were subjected to an evaluation procedure. To efficiently and rapidly progress from newly synthesized copolymers to a lead candidate, we introduced a rational step-by-step screening approach defining key parameters of different complexities (Figure 1). This includes quality measures for the chemical synthesis, information on colloidal stability, and an in vitro evaluation with respect to transfection efficiency and cytotoxicity. By this screening process, we could eliminate polymeric carriers that displayed unfavorable properties while focusing on candidates with favorable properties for in vivo gene delivery. We could identify two key parameters, which were indispensable for polyplex characterization and performance evaluation, namely colloidal stability and the combined transfection value Luc × GFP normalized to dCS c/p 32. The colloidal stability combines key parameters of the polyplex, including polyplex stability, ζ-potential, hydrodynamic diameter, and PDI. The resulting value displays an optimized copolymer/DNA ratio that reflects the lowest amount of derivative necessary to have favorable polyplex characteristics, which is of importance since excessive copolymer amounts are the main source for cytotoxic effects. The merged transfection value Luc × GFP gives a complementary view on transfection efficiency by combining two reporter gene systems to assess on how numerous and how efficient transgenic DNA is delivered to cells. In general, we observed that BPEI derivatives were only effective at higher copolymer/DNA ratios, which is however associated with pronounced cytotoxic effects (data not shown). The toxic effects exerted by the copolymer are linked to the charge density, which is higher for BPEI than for LPEI derivatives [21]. Hence, shielding the charge with PEG moieties has been described as a promising strategy [25,26,27]. However, the conjugation of PEG to our systems led to significant reduction of DNA complexation as well as transfection properties. In contrast, other groups were successful in obtaining effective transfection with similar delivery vectors conjugated to PEG [50,62,63]. This might be due to several reasons such as steric hindrance of different PEG moieties with varying GD that can hamper proper DNA condensation.

The polymeric delivery vector screening provided a first indication of the superior transfection performance of the selected lead candidate, i.e., **dCS-Suc-LPEI-14**. Indeed, follow-up experiments with the scaled-up lead candidate revealed an excellent match between in vitro performance and transfection capacity in vivo. In addition, **dCS-Suc-LPEI-14** showed a good balance between transfection efficiency and cytotoxicity (Figure 4). It should be noted that although the cell viability was part of our screening in cell culture models, this parameter has a limited predictive value towards in vivo toxicity affecting the whole organism. Nevertheless, it facilitates a universal approach that can be easily adapted and applied to similar delivery vectors, enabling the possibility to identify promising lead candidates.

Subsequent to the in vitro evaluation, the lead candidate **dCS-Suc-LPEI-14** was tested in vivo to confirm the validity of our approach. For the experiments in mice, the commercial transfection reagent in vivo-jetPEI was used as a reference. Furthermore, the experiments were carried out with and without dexamethasone pretreatment [64,65] to suppress proinflammatory processes as we observed liver toxicity as indicated by serum markers and jaundice. Dexamethasone premedication is a standard procedure in clinical trials with test medications such as lipid-based delivery systems where adverse immune events or infusion-related reactions have to be avoided [66]. Indeed, pretreatment with dexamethasone was clearly beneficial with respect to a reduction of ALT and both bilirubin markers but had no statistically significant impact on transgene expression (Figure 5c,d). Our non-integrating nanovector-DNA, under the control of a liver-specific P3 promoter [67,68,69] and coding for luciferase, was administered by retrograde intrabiliary infusion. This is an established and efficient way to deliver polymeric delivery vectors to the liver in rodents [70], and is in contrast to a systemic administration [70,71]. Direct intrabiliary infusion allows substantial reduction of the amounts of DNA and polyplexes required. In these experiments only 1 µg nanovector-DNA was administered.

Intrabiliary infusion of **dCS-Suc-LPEI-14** polyplexes resulted in a luciferase transgene expression that was 10-fold higher as compared to in vivo-jetPEI. In addition, our dCS-PEI derivative showed reduced liver toxicity as compared to in vivo-jetPEI. We conclude from this successful proof-of-concept in vivo experiment that covalent combination of dCS and PEI, as realized in the lead compound **dCS-Suc-LPEI-14**, is a promising gene delivery strategy. It combines colloidal stability with nucleic acid condensation ability, biocompatibility, and high transfection efficiency in vitro and in vivo.

## 4. Materials and Methods

### 4.1. Materials and Cell Cultures

Reagents and solvents were purchased from commercial sources (Alfa Aesar, Acros, Fluka, Roth, Polysciences Inc., Sigma Aldrich, TCI, ABCR) and were used without further purification unless otherwise stated. In particular, CS was obtained from TCI (5–20 mPa·s, 0.5% in 0.5% acetic acid at 20 °C; DD: 80%; mixture of three distributions with Mw 478,900, 88,410 and 44,800 Da). BPEI was obtained from ABCR (purity > 99.9%; 1800 Da) and LPEI was obtained from Polysciences Inc. (2500 Da). HCl.NH_2_-PEG-SH (2000 Da) was obtained from Sigma Aldrich. Centrifugations were performed in an Allegra X-30R Centrifuge (Swinging-bucket rotor, 4700 rpm, Beckman Coulter). Dialysis purifications were performed against distilled water at rt unless stated otherwise. Three types of dialysis membranes were used: MWCO 14 kDa (Roth, Membra Cell, regenerated cellulose (RC), dry packaged, treated with glycerine), MWCO 3.5 kDa (Spectra/Por^®^3 RC, dry packaged, glycerine-free), and MWCO 7 kDa (Zellu Trans 6–8 kDa, RC, dry packaged). Average time of dialysis was set to 3 days with water being renewed 3 times per day. Samples were lyophilized in a VaCo 5 Zirbus technology freeze-dryer (0.3 mbar, −80 °C).

Nanovector-DNA were purchased from Nature Technology Corporation (Lincoln, NE, USA). Nanovector-DNA n.P3Luc1 (2951 bp) and n.CAGLuc2 (5700 bp) encode both the firefly luciferase transgene driven by a synthetic liver specific promoter P3 [67,68] and the ubiquitously expressing cytomegalovirus (CMV) enhancer fused to the chicken beta-actin (CAG) promoter, respectively. Nanovector-DNA n.CAG.GFP1 (5200 bp) consists of the CAG promoter and the green florescent protein (GFP) as the reporter transgene.

HuH-7 cells were obtained from RIKEN Cell Bank (Ibaraki, Japan) and cultured at 37 °C with 5% CO_2_ and saturated humidity. Cells were kept in Dulbecco’s modified Eagle’s medium (DMEM) high glucose (4500 mg/L) and additionally supplemented with 10% fetal calf serum FCS and 1% penicillin (10,000 IU/mL)-streptomycin (10,000 μg/mL). Subculturing was performed when cells reached 70% confluency.

### 4.2. Polymers Analytics

^1^H NMR and 2D-DOSY NMR spectra were recorded at rt on Bruker 400 Ultrashield™ Plus and Bruker Avance III HD-600 (respectively 400 and 600 MHz, Bruker, Billerica, MA, USA). Spectra were analyzed using Mestre Nova software. Chemical shifts (δ) were reported in parts per million (ppm) relative to residual solvent peaks rounded to the nearest 0.01 ppm (ref: CD_3_COOD 2.05 ppm and D_2_O 4.79 ppm). Peak multiplicities were indicated as follows: s (singlet), d (doublet), t (triplet), m (multiplet), and br (broad). All integrations were performed per unit of CS. For infrared spectroscopy data, a Perkin Elmer Frontier FTIR system was used with a QUEST ATR Accessory (diamond Ext Range Accy Frontier). GPC analyses were recorded on two different setups: (1): PL-GPC 50 integrated GPC/SEC system (Agilent) equipped with a refractive index detector, a PSS NOVEMA MAX columns set (1× guard column, 10 µm; 2× analytical columns 1000 Å, 10 µm; 1× analytical column 30 Å, 10 µm), using 0.3 M aqueous acetic acid and 0.2 M aqueous sodium acetate as eluents. Samples of CS and dCS (2 mg each) were dissolved overnight in 1.5 mL of mobile phase. The solutions were filtered through a sterile 0.22 µm PTFE filter followed by injection (100 µL). Flow rate of analysis: 1 mL/min at 40 °C. (2): 1260 Infinity II Agilent GPC/SEC system equipped with a Wyatt triple detection setup: multi-angle light scattering (MALS), viscometer, and refractive index detector (DAWN8). Samples were separated using a guard column (OHpak SB-G 6B, 6 × 50 mm) and a mixed-gel column (OHpak SB-806M HQ, 8 × 300 mm). Samples of 10 mg were dissolved for 1 h in 1 mL of acetate buffer (0.3 M aqueous acetic acid and 0.2 M aqueous sodium acetate), followed by filtration through a 0.45 µm PES membrane. The samples were injected (100 µL) into the GPC at a flow rate of 1 mL/min at 30 °C. Runtime was 40 min. Pullulan P20 (180–1,220,000 Da) was used as a reference and stability control. Data were analyzed on Astra software. When necessary, data fitting was performed using exponential degree 2 and forward extrapolation.

### 4.3. Synthesis of Polymers

**Preparation of dCS**: Commercial CS (200 mg) was suspended in a microwave vial containing 20 mL of 1 M HCl aqueous solution, resulting in a CS solution at 1% *w*/*v*. The vial was placed in the microwave reactor and the depolymerization program was started under a constant stirring at 300 rpm. Video monitoring of the solution showed that the sample was entirely solubilized after 1 min (T °C reaching 60–70 °C). The depolymerization program was composed of the following steps: heat as fast as possible to 100 °C, hold at 100 °C for 19 min, cool down to 35 °C as fast as possible. Once the dCS solution reached 30 °C, the solution was neutralized in two steps: first a 10 M NaOH aqueous solution was added dropwise until the solution started to become blurry and reached pH 6.7 (pH-meter monitoring). The suspension was left to equilibrate at rt for 15 min before being centrifuged (4700 rpm, 20 °C, 10 min). Only the liquid fraction was recovered. Its pH was then further increased to 7.0 with dropwise addition of a 0.05 M NaOH aqueous solution. The white suspension was centrifuged a second time under the same conditions to remove the second precipitate, if any. The resulting liquid part was transferred to a dialysis membrane (MWCO 3.5 kDa) and dialyzed against water for 3 days. After freeze drying, a white aerated solid was obtained (25 mg, DD = 79%, Mw = 8.3 kDa). ^1^H NMR (400 MHz, D_2_O/CD_3_COOD 1/1): δ 4.94 (d, J = 8.6 Hz, 0.79H, -OC*H*-O-), 4.63 (br, 0.21H, -OC*H*-O-), 4.10–3.48 (m, 5H), 3.25 (t, J = 9.0 Hz, 1H, -C*H*NH_2_). ^1^H NMR (400 MHz, D_2_O): δ 4.59 (br, 1H, -OC*H*-O-), 4.08–3.42 (m, 5H), 2.85 (br, 1H, -C*H*NH-), 2.07 (s, 3H, -NHCOC*H*_3_). IR (cm^−1^): 3292, 2876, 1644, 1376, 1320, 1151, 1062, 1030, 896, 666.

**Synthesis of dCS-Suc**: dCS (DD = 79%, 1.00 g, 4.72 mmol of reactive glucosamine units, 1.00 equiv.) was dissolved in water (100 mL) at rt and further acidified with HCl 37% aqueous solution (0.300 mL added dropwise to reach complete dissolution). To this solution were slowly added pyridine (5.70 mL, 70.8 mmol, and 15.0 equiv.) and succinic anhydride (477 mg, 4.77 mmol, and 1.01 equiv.). After 20 min, dissolution was complete and pH of the solution was controlled at 7. After 1 h of additional stirring at rt, the mixture was transferred to a dialysis tube (MWCO 3.5 kDa) and dialyzed against water for 3 days. After centrifugation (4700 rpm, 19 °C, and 10 min), the supernatant was recovered and lyophilized. dCS-Suc was obtained as a white aerated solid (913 mg, GD_Suc_ = 63%). ^1^H NMR (600 MHz, D_2_O/CD_3_COOD 1/1): δ 5.20 (br, 0.05H, -OC*H*-O-), 4.94 (br, 0.16H, -OC*H*-O-), 4.59 (br, 0.79H, -OC*H*-O-), 4.04–3.47 (m, 5H), 3.21 (br, 1H, -C*H*NH-), 2.66 (br, 4H, -CH_2_C*H_2_*CO). ^1^H NMR (400 MHz, D_2_O): δ 5.19 (br, 0.05H, -OC*H*-O-), 4.88 (br, 0.16H, -OC*H*-O-), 4.58 (br, 0.79H, -OC*H*-O), 3.98–3.45 (m, 5H), 3.16 (br, 1H, -C*H*NH-), 2.58 (br, 4H, -CH_2_C*H_2_*CO), 2.05 (br, 3H, -NHCOC*H_3_*). IR (cm^−1^): 3270, 2930, 1645, 1549, 1378, 1154, 1064, 1029, 898, 622.

**Synthesis of dCS-Suc-BPEI**: dCS-Suc (DD = 83%, GD_Suc_ = 45%, 200 mg, 1.03 mmol of reactive units, and 1.00 equiv.) was dissolved in water (10 mL) for 15 min at rt. DMTMM (285 mg, 1.03 mmol, and 1.00 equiv.) was added to this solution, which was stirred for 10 min at rt. To the clear solution was added dropwise a solution of BPEI (1.8 kDa, 2.87 g, 0.638 mmol, and 0.620 equiv.) dissolved in 10 mL of water. After 3 h of stirring, the very clear reaction mixture was transferred to a dialysis tube (MWCO 14 kDa) and dialyzed against water for 3 days. After centrifugation (4700 rpm, 18 °C, and 10 min), the supernatant was recovered and lyophilized. dCS-Suc-BPEI was obtained as a white aerated solid (650 mg, GD_BPEI_ = 18%). ^1^H NMR (400 MHz, D_2_O/CD_3_COOD 1:1): δ 5.25 (br, 0.05H, -OC*H*-O-), 4.94 (br, 0.38H, -OC*H*-O-), 4.60 (br, 0.57H, -OC*H*-O-), 4.13–2.75 (m, 36H), 2.69 (br, 4H, -CH_2_C*H*_2_CO). ^1^H NMR (400 MHz, D_2_O): δ 4.58 (br, 1H, -OC*H*-O-), 4.01–3.08 (m, 6H), 3.07–2.47 (br, 30H), 2.06 (s, 3H, NHCOC*H*_3_).

**Synthesis of dCS-Suc-LPEI**: dCS-Suc (DD = 79%, GD_Suc_ = 63%, 200 mg, 0.542 mmol of reactive units, and 1.00 equiv.) was dissolved in water (20 mL) over 15 min from rt to 50 °C. DMTMM (150 mg, 0.542 mmol, and 1.00 equiv.) was added in one portion, followed by heating of the reaction mixture at 60 °C for 10 min (clear solution). LPEI (677.5 mg, 0.271 mmol, and 0.50 equiv.) was added in 4 equal portions and under vigorous stirring (>500 rpm). pH was controlled at 10. After 3 h of heating at 60 °C under vigorous stirring, the mixture was cooled down and split into 6 dialysis tubes (MWCO 7 kDa). They were filled with additional water (30 mL) and dialyzed against water for 3 days. After centrifugation (4700 rpm, 18 °C, and 10 min), the supernatant was recovered and lyophilized. dCS-Suc-LPEI was obtained as of a white aerated solid (135 mg, GD_LPEI_ = 14%). ^1^H NMR (600 MHz, D_2_O/CD_3_COOD 1/1): δ 4.93 (br, 0.16H, -OC*H*-O-), 4.60 (br, 0.84H, -OC*H*-O-), 4.14–2.96 (m, 39H), 2.66 (br, 4H, -CH_2_C*H_2_*CO). ^1^H NMR (400 MHz, D_2_O): δ 4.59 (br, 0.84H, -OC*H*-O-), 4.48 (br, 0.16H, -OC*H*-O-), 4.12–2.63 (m, 39H), 2.62–2.44 (br, 4H, -CH_2_C*H_2_*CO), 2.06 (s, 3H, NHCOC*H_3_*). IR (cm^−1^): 3272, 2917, 2847, 1651, 1555, 1464, 1408, 1372, 1303, 1109, 1063, 1030, 899, 812, 646.

**Synthesis of dCS-NSucBPEI-OPEG-SH**: dCS-Suc-BPEI (DD = 80%, GD_Suc_ = 47%, GD_BPEI_ = 11%, 30.0 mg, 0.0724 mmol, and 1.00 equiv.) was dissolved in dry DMSO (2.5 mL) over 15 min at rt. After addition of CDI (12.0 mg, 0.0724 mmol, and 1.00 equiv.), the white solution was stirred at rt for 30 min. A solution of PEG (2 kDa, 29 mg, 0.0145 mmol, and 0.200 equiv.) in 0.5 mL of DMSO was then added dropwise. The reaction mixture was stirred at rt for 19 h, before being dialyzed against water for 3 days (MWCO 14 kDa). The solution remained homogeneous over time. It was then centrifuged (4700 rpm, 18 °C, and 10 min) and the supernatant was recovered. dCS-NSucBPEI-OPEG-SH was obtained after freeze drying as a white aerated solid (37.3 mg, GD_PEG_ = 26%). ^1^H NMR (600 MHz, D_2_O/CD_3_COOD 1:1): δ 4.94 (br, 0.33H, -OC*H*-O-), 4.60 (br, 0.67H, -OC*H*-O-), 4.02–2.74 (m, 70H), 2.66 (br, 4H, -CH_2_C*H*_2_CO). ^1^H NMR (400 MHz, D_2_O): δ 4.65–4.43 (br, 1H, -OC*H*-O-), 4.00–2.41 (m, 66H), 2.06 (s, 3H, NHCOC*H*_3_).

**Synthesis of dCS-NSuc-OPEG-SH**: To a solution of dCS-Suc (DD = 85%, GD_Suc_ = 44%, 100 mg, 0.473 mmol of reactive units, and 1.00 equiv.) in 6 mL of dry DMSO, was added at rt and in one portion CDI (77 mg, 0.473 mmol, and 1.00 equiv.). The white homogenous mixture was stirred at rt for 30 min. DIPEA (41 μL, 0.236 mmol, and 0.500 equiv.) was then added to the reaction mixture, followed by a dropwise addition of a solution of PEG (2 kDa, 194 mg, 0.0946 mmol, and 0.200 equiv.) in DMSO (4 mL). After 17 h of stirring at rt, the white mixture was transferred to a dialysis tube (MWCO 7 kDa) and dialyzed against water for 3 days. The solution was centrifuged (4700 rpm, 21 °C, and 10 min) and the supernatant was lyophilized. dCS-NSuc-OPEG-SH was obtained as a white and aerated solid (200 mg, GD_PEG_ = 14%). ^1^H NMR (400 MHz, D_2_O/CD_3_COOD 1:1): δ 4.93 (br, 0.41H, OC*H*-O-), 4.60 (br, 0.59H, OC*H*-O-), 4.18–3.03 (m, 30H), 2.94 (t, 2H, -C*H*_2_CH_2_SH), 2.66 (br, 4H, -CH_2_C*H*_2_CO).

**Synthesis of dCS-NSucLPEI-OPEG-SH**: To a clear solution of dCS-NSuc-OPEG-SH (DD = 85%, GD_Suc_ = 44%, GD_PEG_ = 14%, 50.0 mg, 0.100 mmol, and 1.00 equiv.) 8 mL of water was added at rt and in one portion DMTMM (13.9 mg, 0.050 mmol, and 0.500 equiv.). After 15 min of stirring from rt to 60 °C, LPEI (2.5 kDa, 125 mg, 0.050 mmol, and 0.500 equiv.) was added to the reaction mixture in 4 portions. Finally, DIPEA (3.5 μL, 0.020 mmol, 0.200 equiv.) was added and the reaction mixture was stirred at 60 °C for 3 h. After cooling down, the homogenous mixture was split into two dialysis tubes (MWCO 7 kDa) and dialyzed against water. The solution was centrifuged (4700 rpm, 20 °C, and 10 min), and the supernatant was freeze dried to yield dCS-NSucLPEI-OPEG-SH as a white aerated solid (37.0 mg, GD_LPEI_ = 22%). ^1^H NMR (600 MHz, D_2_O/CD_3_COOD 1:1): δ 4.95 (br, 0.41H, OC*H*-O-), 4.61 (br, 0.59H, OC*H*-O-), 4.12–3.20 (m, 86H), 2.67 (br, 4H, -CH_2_C*H*_2_CO).

### 4.4. Polymer Complexation with DNA

The dCS-Suc-PEI copolymers were reconstituted in purified water and supported with HCl, when required, to a minimum pH of four. For used concentration refer to Table 2. The polymer was diluted in glucose solution and added to the DNA, vortexed, resulting in a final 5% (*w*/*v*) glucose solution, before incubating for 15 min at room temperature. The ability of polymers to condensate DNA was confirmed by agarose gel electrophoresis. To 20 μL polyplexes at various c/p ratios with a final concentration of 15 μg DNA/mL, 4 μL DNA sample-buffer was added (15% Ficoll400 and 0.1% Bromophenol blue) and administered to electrophoresis on a 0.7% agarose gel (including 1 μg/mL ethidium bromide) during 90 min at an electric potential of 90 V in tris-borate-EDTA buffer (TBE). A molecular-weight size marker (Precision Plus Protein Dual Color Standards, Bio-Rad, Hércules, CA, USA) was included as reference. DNA bands were imaged with the ChemiDoc MP Imaging system using Image lab V6.1 software (Bio-Rad).

To evaluate the accessibility of complexed DNA, polyplex solutions at various c/p ratios were added to 20 μg DNA/mL and RedSafe nucleic acid staining solution (1:66,666, iNtRON Biotechnology, Sagimakgol-ro, Korea). Fluorescence emission was measured using an UV/VIS microplate reader (Ex: 309 nm, Em: 537 nm). Results were normalized to free DNA.

### 4.5. Colloidal Stability

Hydrodynamic diameter D and polydispersity index (PDI) were determined by dynamic light scattering (DLS) and ζ-potential was determined by electrophoretic light scattering using Zetasizer Nano-ZS Zen3600 and Zetasizer Ultra with the Zetasizer DTS and ZS explorer software, respectively (Malvern Panalytical, Worcestershire, UK). For ζ-potential measurements, polyplexes were diluted in a 5% glucose solution.

### 4.6. Transfection

Transfection experiments were assessed in 24-multiwell plates with 2 × 10^4^ HuH-7 cells/well. Subsequently, 100 μL polymer solution was added per well at a final concentration of 10 μg DNA/mL.

Luciferin assay was conducted to determine transgene expression. Forty-eight hours after addition of polyplex solution encoding firefly luciferase, cells were washed with phosphate-buffered saline (PBS) and lysed with 60 μL luciferase cell culture lysis reagent (25 mM Tris-phosphate pH 7.8, 2 mM DTT, 2 mM DCTA, 50% glycerol, 5% Triton X-100) on ice for 15 min. Cell lysate was centrifuged (21,000× *g*, 3 min) and 20 μL supernatant was measured using a bioluminescence microplate reader after addition of 100 μL D-luciferin solution (570 µM). Transfection experiments were normalized to protein concentration of cell lysate supernatants. Protein concentration was quantified by UV-absorption at 280 nm.

Quantification of delivered DNA was conducted by quantitative polymerase-chain reaction (qPCR). Twenty-four hours after addition of polyplex solution with nanovector-DNA encoding firefly luciferase, transfected DNA was extracted using QIAprep Spin Miniprep Kit (Quiagen, Venlo, The Netherlands) according to the manufacturer’s protocol. Subsequently, qPCR was performed using KAPA SYBR Fast qPCR Master Mix (KAPA Biosystems, Wilmington, MA, USA), equal voluminal of isolated DNA, and the following primers: Luciferase forward 5′-AACAGGTTGAACTGCTGATCC-3′ and Luciferase reverse 5′-ACAAGATGTGCGAACTCGATATT-3′. qPCR reactions were performed applying 40 cycles of 95 °C for 15 s and 60 °C for 1 min, using a Rotor-Gene Cycler (Corbett research, Sydney, New South Wales, Australia). Readout data were normalized to the DNA calibration curve.

The number of transfected cells was quantified using flow cytometry. Forty-eight hours after addition of the polyplex solution encoding eGFP, cells were trypsinized and resuspended in FACS-Buffer (1% FCS, 2.5 mM EDTA, 0.05% NaN_3_ in PBS) containing 7 aminoactinomycin D (7-AAD, 2 μg/mL) and annexin V (1 μg/mL). For the flow cytometry, singlets, 7 AAD, and Annexin V negative cells were gated and analyzed for eGFP expression. A total of 10,000 cells per sample was analyzed using FACS Canto II (BD Bioscience, San Jose, CA, USA) and data was subsequently processed using FlowJo software (TreeStar, Ashland, OR, USA).

### 4.7. Confocal Microscopy

HuH-7 cells were cultured in Ibidi μ–Slide (Ibidi, Martinsried, Germany). Forty-eight hours after addition of polyplex solution encoding eGFP by nanovector-DNAs, cell nuclei were stained using Hoechst-33342 (2 μg/mL) in D-PBS for 10 min at 37 °C and thereafter incubated in fresh media. Samples were analyzed on an Olympus FluoView3000 inverted confocal microscope (Olympus, Tokyo, Japan) with an UPLSAPO 30× silicon oil-immersion objective (NA 1.05). Images were further processed using Olympus FV31S-SW (Olympus) and Omero version 5.4.10 software [72].

### 4.8. Cytotoxicity

Cytotoxicity of polyplexes was assessed by a cell viability assay. On a 96-multiwell plate 1.5 × 103 HuH-7 cells/well were seeded and polyplexes of 10 μg DNA/mL with various c/p ratios were added to fresh media. Terfenadine (1–10 nM) was administered as a negative control. Following 48 h incubation, 20 μL of MTS reagent was added to each well and incubated for 2 h at 37 °C. Conversion of MTS by living cells to a metabolite was monitored by a colorimetric reaction at 490 nm using an UV/VIS microplate reader.

### 4.9. Retrograde Intrabiliary Infusion in Mice

Animal experiments conducted under license ZH082/19 (31232), were approved by the State Veterinary Office of Zürich (approved on 29 August 2019) and carried out according to the guidelines of the Swiss Law of Animal Protection, the Swiss Federal Act on Animal Protection (1978), and the Swiss Animal Protection Ordinance (1981). Wild-type C57BL/6JRccHsd male mice aged 6–8 weeks (20–24 g) were obtained from Envigo, Horst, Netherlands. Mice were maintained under a 12-h dark–light cycle at a standardized environment with controlled humidity and temperature. They had ad libitum access to standard chow and water. For pain relief, mice received a combination of 0.1 mg/kg buprenorphine (Temgesic, Indivior Schweiz AG, Baar, Switzerland) and 5 mg/kg carprofen (Rimadyl, Pfizer, New York, NY, USA) subcutaneously 30 min before and after surgery. For anesthesia, a nose mask was fixed with continued treatment of 2–3% isoflurane. The surgical intervention was adapted from Berntsen et al. [73]. Following a midline laparotomy, polyethylene tubing (Smiths Medical, Adliswil, Switzerland) was carefully inserted into the gallbladder and placed just proximal to the junction of the cystic duct. A silk tie was used to secure the infusion tubing. The common bile duct was obstructed by a micro vessel clamp (60 g; Fine Science Tools, Heidelberg, Germany) to ensure fluid-injection directly into the liver and at the same time to prevent fluid from entering the pancreas and duodenum. A total volume of 400 µL containing either in vivo-jetPEI at c/p 1.1 (Polyplus, Illkirch, France) or dCS-Suc-LPEI-14 at c/p 2 (batch #LN276), each encapsulating 1 µg of nanovector-DNA n.P3Luc1 in 5% glucose solution, was administered. Duration of infusion was 10 min using an infusion pump (Harvard Apparatus, Holliston, MA, USA) and a normo-dynamic infusion rate of 0.04 mL/min. After infusion, the micro vessel clamp was removed from the common bile duct. The infusion tubing was withdrawn from the gallbladder, the cystic duct was ligated, and a cholecystectomy was performed. To suppress the inflammatory response, a single dose of dexamethasone (10 mg/kg) (Mephameson, Mepha Pharma AG, Basel, Switzerland) was infused i.p. 15 h prior to surgical intervention as indicated.

### 4.10. In Vivo Imaging of Gene Expression and Assessment of Liver Specific Toxicity

Mice were i.p. injected with 150 mg/kg D-luciferin potassium salt (Gold Biotechnology, St. Louis, MO, USA). After D-luciferin injection, bioluminescence was monitored using a bioimaging system (IVIS 200, Perkin Elmer, Santa Clara, CA, USA). Signals were quantified using Living Image 3.2 software (Perkin Elmer). After termination of the experiment on day 3, blood samples were collected from the vena cava. Alanine aminotransferase (ALT), alkaline phosphatase (ALP), and direct/total bilirubin levels in serum samples were analyzed as indicated by the manufacturer (Abbott Alinity C System, Abbot Laboratories, Chicago, IL, USA).

## 5. Conclusions

In the present work, small molecular weight depolymerized CS was used as a platform for sequential and selective conjugation of both amino and hydroxyl functionalities with PEI and PEG derivatives. These cationic copolymers were assessed for their DNA condensation ability and the resulting polyplexes were evaluated with respect to key parameters such as colloidal stability, in vitro and in vivo transfection efficiency and cytotoxicity. We conclude that intrabiliary administration of a non-integrating DNA vector under the control of a liver-specific promoter allows for an efficient transfection of the liver. We propose that the transfection efficiency can be further optimized by functionalization of the polymer conjugates. This could include the conjugation of tissue-specific targeting moieties and cell-penetrating peptides at the surface of the polyplexes—although challenging, this solution would allow systemic administration. Alternatively, intrabiliary injection is a minimal invasive endoscopic procedure, comparable to endoscopic stenting of the bile duct. Upon chemical modification of polyplexes to reduce and minimize toxicity, this route offers interesting options with respect to a future clinical evaluation of the presented non-viral gene therapy approach.

## Figures and Tables

**Table 1:** Physicochemical properties of selected dCS-PEI derivatives and their precursors. Characteristics of the dCS backbone used, grafting degree of the succinyl linker or the polymer on dCS, and water solubility are displayed for each copolymer. Grafting degrees (GD) (%) of LPEI and BPEI are indicated at the end of the name of each functionalized polymer. Molecular weights of the dCS backbones were estimated by gel permeation chromatography (GPC) and GD were determined by ^1^H NMR. Nomenclature: cf. Figure 2.

Polymer	Total Molecular Weight (Da)	Depolymerized Chitosan (dCS)		Succinate (Suc)		Polyethylenimine (PEI)		Polyethylene Glycol (PEG)		Solubility ^1^ (mg/mL)
		Mw (Da)	DD (%)	GD (%)	w%	GD (%)	w%	GD (%)	w%	
**Starting polymer** - *dCS* - *LPEI* - *BPEI*										
	8300	8300	79	-	-	-	-	-	-	3
	2500	-	-	-	-	-	-	-	-	5
	1800	-	-	-	-	-	-	-	-	>10
**BPEI derivatives** - *dCS-Suc-BPEI-11* - *dCS-Suc-BPEI-13* - *dCS-Suc-BPEI-67* **PEG-conjugated BPEI derivatives** - *dCS-NSucBPEI-OPEG-SH*										
	19,100	7800	80	47	12	11	48	-	-	>10
	16,900	6200	80	54	12	13	51	-	-	7.5
	75,300	9000	80	44	3	67	85	-	-	>10
									
	43,500	7800	80	47	5	11	21	26	56	>10
**LPEI derivatives ^2^** - *dCS-Suc-LPEI-11a* - *dCS-Suc-LPEI-11b* - *dCS-Suc-LPEI-14 (scale up)* **PEG-conjugated LPEI derivatives** - *dCS-NSucLPEI-OPEG-SH*										
	16,800	5700	80	51	10	11	56	-	-	>10
	23,800	7800	80	47	10	11	56	-	-	>10
	28,600	8300	79	63	11	14	60	-	-	>10
									
	48,300	7800	85	44	4	22	53	14	27	>10

^1^ The dCS-Suc-PEI copolymers were reconstituted in purified water and supported with HCl, when required, to a minimum pH of 4. ^2^ **dCS-Suc-LPEI-11a** is the result of a non-centrifuged solution whereas **dCS-Suc-LPEI-11b** represents the supernatant of a centrifuged solution of dCS-Suc-LPEI. Every other copolymer detailed in this table is the result of freeze-dried supernatant obtained after centrifugation (post-dialysis).

**Table 2:** Colloidal stability and in vitro performance of DNA complexed dCS-PEI vectors. DNA loading degree is defined by the c/p value (ratio polymer/DNA; %*w*/*w*). Polyplex stability is determined by gel retardation assay. The optimized colloidal stability defines the c/p ratio for their further use in vitro. A polydispersity index (PDI) value equal or below 0.2 is considered to be monodisperse. The in vitro gene expression was determined in the hepatocellular carcinoma cell line HuH-7 using two reporter nanovector-DNA (n.CAGLuc2 and n.CAG.GFP1). Cytotoxicity effects were assessed using a cell viability assay (MTS assay). As a result, **dCS-Suc-LPEI-11b** was selected as lead candidate. Values are means ± SD, *n* ≥ 3. Nomenclature: cf. Figure 2.

Polymers	Polyplex Stability (c/p Ratio)	Optimized Colloidal Stability (c/p Ratio)	ζ-Potential (mV)	Hydrodynamic Diameter D (nm)	PDI	Luc x GFP Normalized to dCS c/p 32 (Fold)	MTS (% Viable Cells)
**Starting polymer** - *dCS* - *LPEI* - *BPEI*							
	32	32	22 ± 4	68 ± 5	0.23 ± 0.03	1 ± 1	111 ± 5
	32	32	28 ± 7	48 ± 1	0.17 ± 0.01	2103 ± 61	25 ± 1
	16	16	21 ± 1	91 ± 1	0.09 ± 0.02	3415 ± 119	43 ± 4
**BPEI derivatives** - *dCS-Suc-BPEI-11* - *dCS-Suc-BPEI-13* - *dCS-Suc-BPEI-67* **PEG-conjugated BPEI derivatives** - *dCS-NSucBPEI-OPEG-SH*							
	1	2	19 ± 3	68 ± 2	0.22 ± 0.01	415 ± 24	81 ± 1
	0.5	4	16 ± 4	62 ± 1	0.22 ± 0.01	1174 ± 6	81 ± 7
	0.5	1	24 ± 2	79 ± 1	0.21 ± 0.01	3488 ± 160	63 ± 2
						
	1	2	13 ± 1	121 ± 1	0.21 ± 0.01	0 ± 0	91 ± 2
**LPEI derivatives** - *dCS-Suc-LPEI-11a* - *dCS-Suc-LPEI-11b* **PEG-conjugated LPEI derivatives** - *dCS-NSucLPEI-OPEG-SH*							
	1	1	17 ± 5	74 ± 2	0.25 ± 0.02	3076 ± 73	97 ± 5
	2	2	23 ± 1	87 ± 1	0.16 ± 0.01	71,567 ± 1592	101 ± 9
						
	2	2	18 ± 1	85 ± 1	0.17 ± 0.01	0 ± 0	91 ± 1

## Data Availability

Raw data will be accessible from zenodo (https://zenodo.org/).

## Supplementary Materials

The following are available online at https://www.mdpi.com/article/10.3390/ijms22083828/s1, Table S1: Screening of coupling agents and reaction conditions for the conjugation of dCS to BPEI, Scheme S1: Synthetic strategies for the preparation of dCS-BPEI conjugates, Figure S1: Detailed representation of PEI conjugation sites to dCS-Suc, Scheme S2: Strategies for the dual functionalization of dCS with PEI and PEG polymers, Table S2: Selection of the optimal synthetic sequence for the functionalization of amino and hydroxyl groups of dCS, NMR analyses and calculations for dCS and derivatives, Table S3: DNA exclusion assay to evaluate accessibility of DNA complexed with polymeric conjugates, Table S4: Scale up and reproducibility of dCS-Suc-LPEI synthesis, Figure S2: Quantitative analysis of in vitro GFP expression of the lead candidate dCS-Suc-LPEI-14.

## Abbreviations

7-AAD	7-Aminoactinomycin D
ALP	Alkaline phosphatase
ALT	Alkaline transaminase
BPEI	Branched polyethylenimine
CDI	1,1′-carbonyldiimidazole
c/p	weight ratio of polymer [c] to nucleic acid [p]
CS	Chitosan
dCS	Depolymerized chitosan
DD	Deacetylation degree
DIPEA	N,N-diisopropylethylamine
DLS	Dynamic light scattering
DMSO	Dimethyl sulfoxide
DMTMM	4-(4,6-dimethoxy-1,3,5-triazin-2-yl)-4-methyl-morpholinium chloride
DNA	Deoxyribonucleic acid
DOSY	Diffusion order spectroscopy
DSC	N-N’ disuccinimidyl carbonate
EDC	1-ethyl-3-(3-dimethylaminopropyl)carbodiimide
ELS	Electrophoretic light scattering
FACS	Fluorescence-activated cell sorting
FTIR	Fourier-transform infrared spectroscopy
GD	Grafting degree
GFP	Green fluorescent protein
GPC	Gel permeation chromatography
IVIS	In vivo Imaging system
LPEI	Linear polyethylenimine
Luc	Luciferase
Mn	Number average molecular weight
Mp	Peak molecular weight
MTS	3-(4,5-dimethylthiazol-2-yl)-5-(3-carboxymethoxyphenyl)-2-(4-sulfophenyl)-2H-tetrazolium
M_W_	Weight average molecular weight
NHS	N-Hydroxysuccinimide
NMR	Nuclear magnetic resonance
NSuc	Suc linked to amine groups
OPEG	PEG linked to hydroxyl groups
PBS	Phosphate buffered saline
PDI	Polydispersity index
PEG	Polyethylene glycol
PEI	Polyethylenimine
pH	Potential of hydrogen
PLL	Poly-L-Lysine
rt	Room temperature
Suc	Succinate
w%	Weight percentage compared to total molecular weight
