# Supplementary material for: Development of Covalent Chitosan-Polyethylenimine Derivatives as Gene Delivery Vehicle: Synthesis, Characterization, and Evaluation
Source: Int J Mol Sci. 2021 Apr 7;22(8):3828. doi: 10.3390/ijms22083828 (PMC8067803; doi:10.3390/ijms22083828)
Supplement: Supplementary file 1 [file ijms-22-03828-s001.pdf]

Supporting Information

Development of Covalent Chitosan-Polyethylenimine Derivatives as Gene Delivery Vehicle: Synthesis, Characterization, and Evaluation

Laura Nicolle [1†], Jens Casper [2†], Melanie Willimann [3†], Céline M.A. Journot [1], Pascal Detampel [2], Tomaž Einfalt [2], Hiu Man Grisch-Chan [3], Beat Thöny [3], Sandrine Gerber-Lemaire [1,*] and Jörg Huwyler [2,*]

[1]Institute of Chemical Sciences and Engineering Ecole Polytechnique Fédérale de Lausanne, Group for Functionalized Biomaterials, EPFL SB ISIC SCI-SB-SG, Station 6, CH-1015 Lausanne, Switzerland; laura.nicolle@epfl.ch (L.N.); celine.journot@epfl.ch (C.M.A.J.); sandrine.gerber@epfl.ch (S.G.-L.)
[2]Division of Pharmaceutical Technology, Department of Pharmaceutical Sciences, University of Basel, Klingelbergstrasse 50/70, 4056 Basel, Switzerland; jens.casper@unibas.ch (J.C.); pascal.detampel@unibas.ch (P.D.); tomaz.einfalt@unibas.ch (T.E.)
[3]Division of Metabolism and Children's Research Center, University Children's Hospital Zurich, 8032 Zürich, Switzerland; HiuMan.Grisch@kispi.uzh.ch (H.M.G.); Melanie.Willimann@kispi.uzh.ch (M.W.); Beat.Thoeny@kispi.uzh.ch (B.T.)
*Correspondence: sandrine.gerber@epfl.ch (S.G.-L.), Tel. :+41 21 693 93 72 ; joerg.huwyler@unibas.ch (J.H.); Tel.: +41 61 207 15 13

† These authors contributed equally to this work

Table of contents

Table S1: Screening of coupling agents and reaction conditions for the conjugation of dCS to BPEI S-3

Scheme S1: Synthetic strategies for the preparation of dCS-BPEI conjugates S-3

Figure S1: Detailed representation of PEI conjugation sites to dCS-Suc S-4

Scheme S2: Strategies for the dual functionalization of dCS with PEI and PEG polymers S-5

Table S2: Selection of the optimal synthetic sequence for the functionalization of amino and hydroxyl groups of dCS S-5

NMR analyses and calculations for dCS and derivatives S-6

- Characterization of dCS S-6
- Characterization of dCS-Suc S-8
- Characterization of dCS-Suc-LPEI S-11
- Characterization of dCS-Suc-BPEI S-14
- Characterization of dCS-NSucBPEI-OPEG-SH S-17
- Characterization of dCS-NSuc-OPEG-SH S-20
- Characterization of dCS-NSucLPEI-OPEG-SH S-23

Table S3: DNA exclusion assay to evaluate accessibility of DNA complexed with polymeric conjugates S-26

Table S4: Scale up and reproducibility of dCS-Suc-LPEI synthesis S-26

Figure S2: Quantitative analysis of *in vitro* GFP expression of the lead candidate dCS-Suc-LPEI-14 S-27

Table S1. Screening of coupling agents and reaction conditions for the conjugation of dCS to BPEI (1.2 kDa, 50 wt% aq. solution).

Starting material	Coupling agent	Reaction conditions[1]	Comments
dCS	CDI	DMSO, 80 °C, 30 min, then BPEI, 80 °C, 18 h	Low grafting
dCS	DSC	PBS, 80 °C, 18 h then BPEI, H_2O, 80 °C, 17 h	Poor reactivity of dCS-DSC intermediate
dCS-Suc	DMTMM	DMTMM, BPEI, DIPEA, H_2O, rt, 3 h	Covalent grafting
dCS-Suc	EDC/sulfo-NHS	DIPEA, H_2O, rt, 1 h then BPEI, rt, 24 h	Mixture of covalent and non-covalent conjugates

[1]For this screening, BPEI 1.2 kDa was used instead of BPEI 1.8 kDa. BPEI was obtained from Sigma Aldrich (solution 50 wt% in H_2O, Mn ≈ 1'200 Da, Mw ≈ 1'300 Da, determined by light scattering). CDI: 1,1'-carbonyldiimidazole; DSC: N,N'-disuccinimidyl carbonate; DMTMM: 4-(4,6-dimethoxy-1,3,5-triazin-2-yl)-4-methyl-morpholinium chloride; EDC: 1-ethyl-3-carbodiimide hydrochloride; sulfo-NHS: N-hydroxysulfosuccinimide.

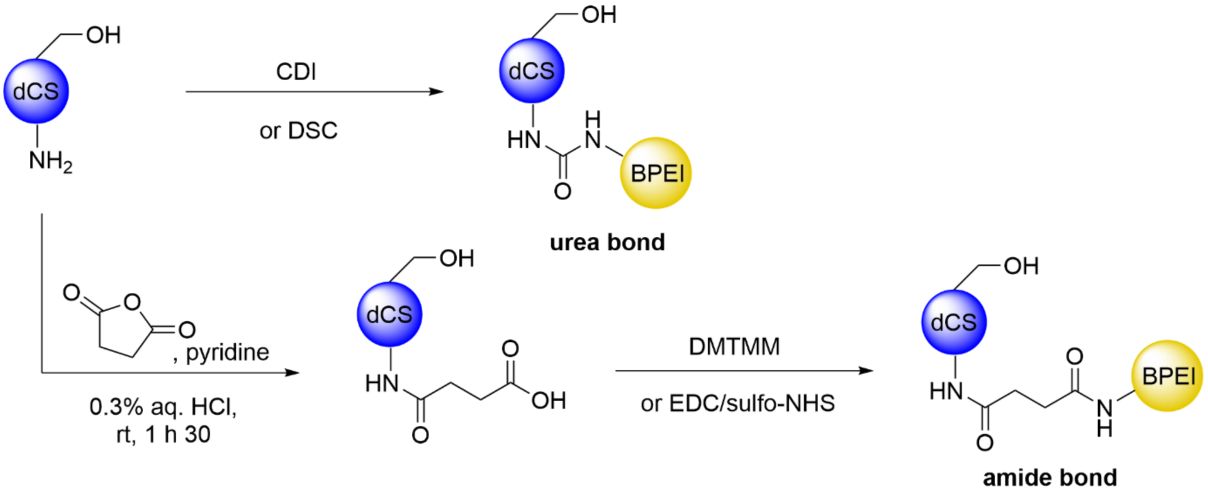

Scheme S1. Synthetic strategies for the preparation of dCS-BPEI conjugates.

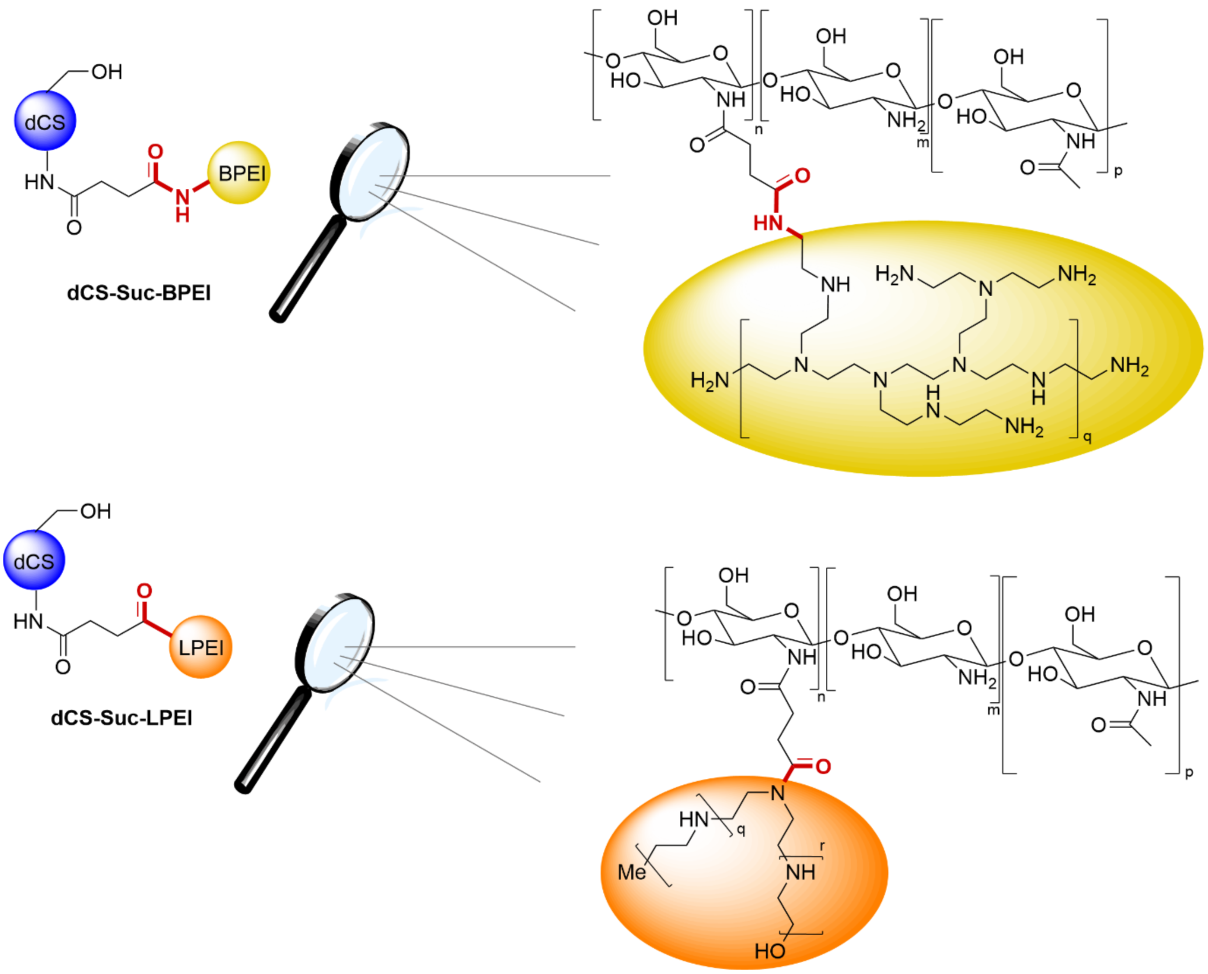

Figure S1. Detailed representation of PEI conjugation sites to dCS-Suc. Both BPEI and LPEI bind to dCS-Suc *via* an amide bond. LPEI contains only secondary amines, therefore only tertiary amides can be formed, whereas BPEI can form secondary and tertiary amides thanks to its primary and secondary amines (right side of the Figure). In order to have an appropriate representation for each product, it was decided to draw a simplified structure (left side of the Figure) for both of them, although both PEI form amide bonds with the succinyl linker.

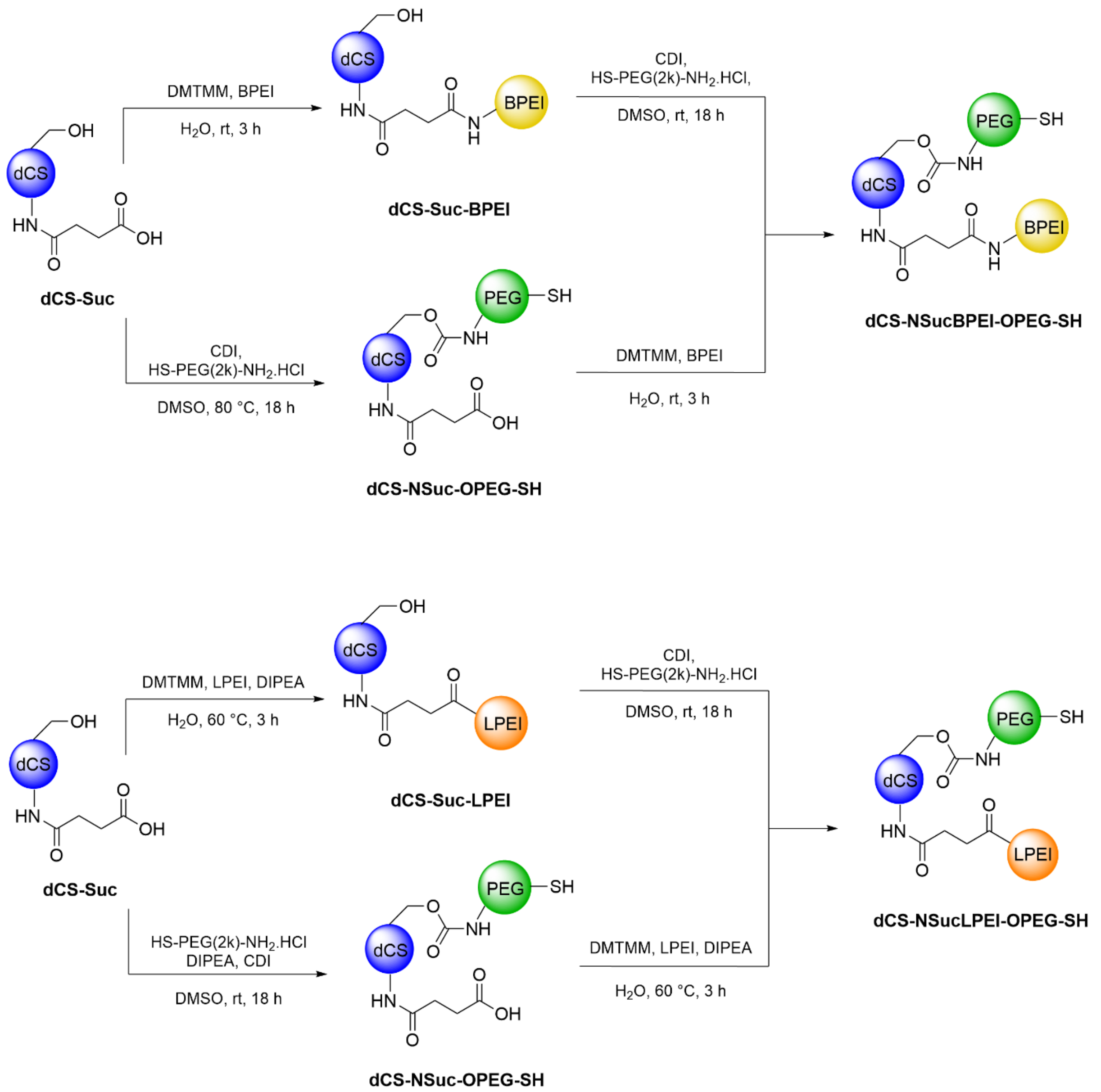

Scheme S2. Strategies for the dual functionalization of dCS with PEI and PEG polymers

Table S2. Selection of the optimal synthetic sequence for the functionalization of dCS amino and hydroxyl functionalities.

Derivative	Order of addition	Result[1]
dCS-NSucBPEI-OPEG-SH	*Step 1:* BPEI – *Step 2:* PEG	Covalent dual grafting
	Step 1: PEG – *Step 2:* BPEI	Mixture of covalent and non-covalent conjugates
dCS-NSucLPEI-OPEG-SH	*Step 1:* LPEI – *Step 2:* PEG	Mixture of covalent and non-covalent conjugates
	Step 1: PEG – *Step 2:* LPEI	Covalent dual grafting

[1]The final conjugates were analyzed by ^{1}H and 2D-DOSY NMR experiments. Fully covalent derivatives were identified by the alignment of all diffusion constants in the 2D-DOSY spectrum.

NMR analyses and calculations for dCS and derivatives

All synthesized derivatives were characterized by ^{1}H and 2D-DOSY NMR. GD values were determined from ^{1}H NMR spectra. For each conjugate, the following characteristics were estimated: grafting degree (GD), average molecular weight (MW).

Characterization of dCS

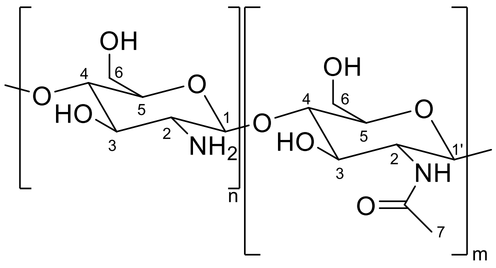

^{1}H NMR (400 MHz, D_2O/CD_3COOD 1/1): δ 4.94 (d, J = 8.6 Hz, 0.79H, H1), 4.63 (br, 0.21H, H1’) 4.10 – 3.48 (m, 5H, H3-4-5-6), 3.25 (t, J = 9.0 Hz, 1H, H2).

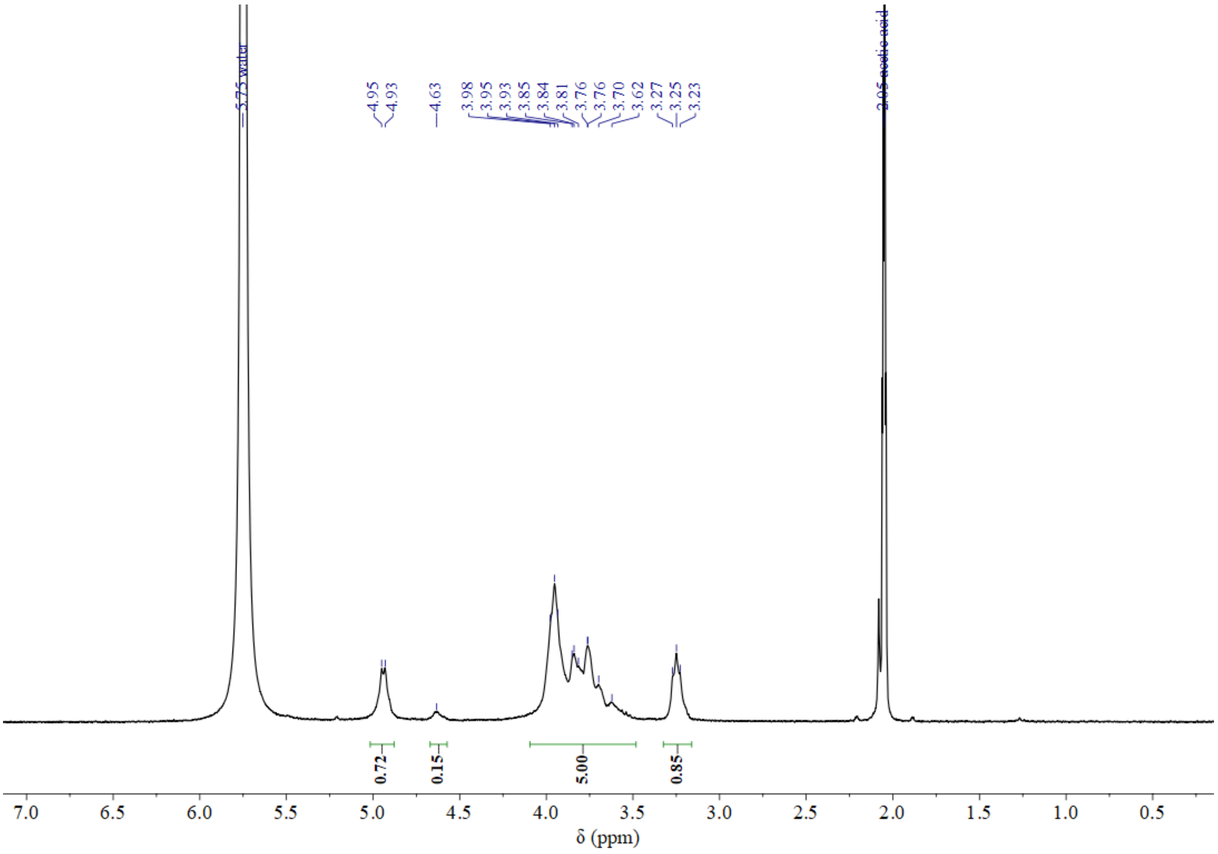

^1H NMR (400 MHz, D_2O): δ 4.59 (br, 1H, H1 + H1'), 4.08 – 3.42 (m, 5H, H3-4-5-6), 2.85 (br, 1H, H2), 2.07 (s, 3H, H7).

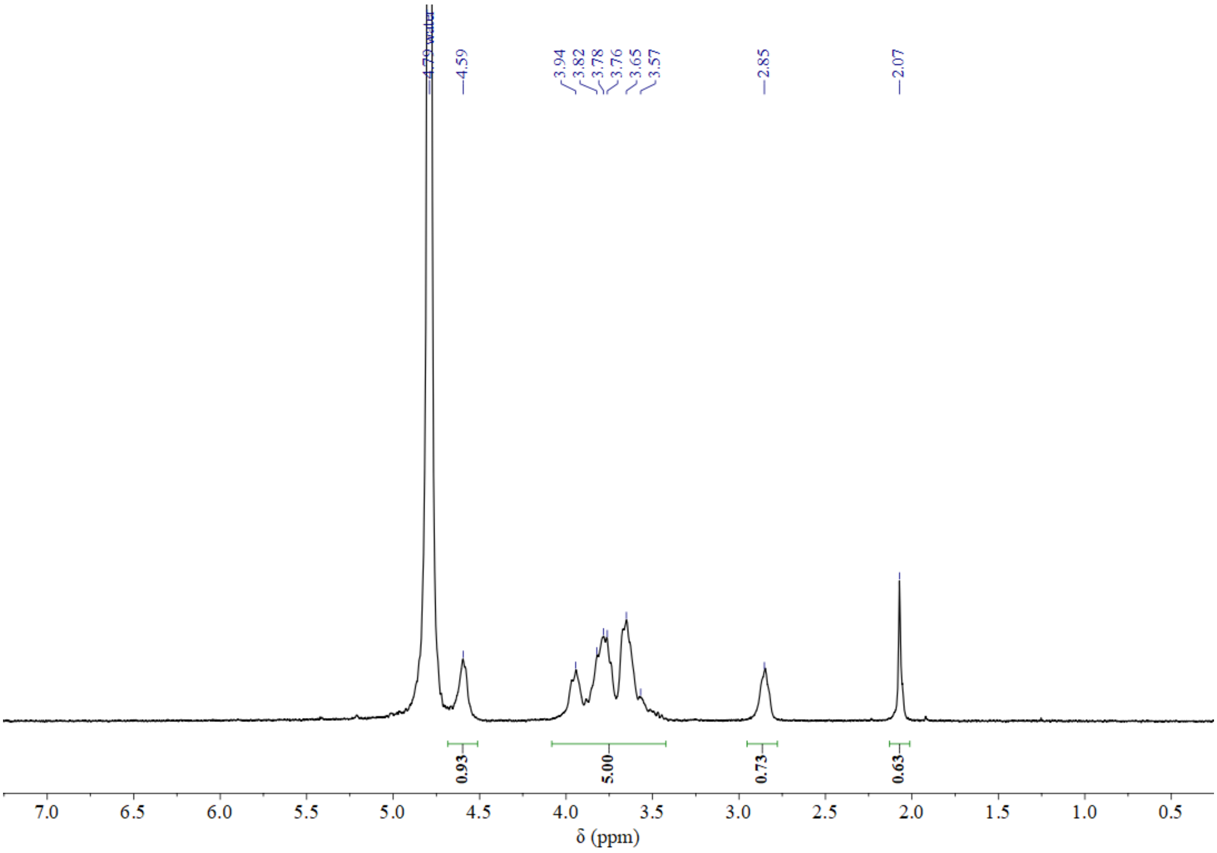

IR (cm^{-1}): 3292, 2876, 1644, 1376, 1320, 1151, 1062, 1029, 896, 666.

Determination of the molecular weight by GPC

Mp (kDa)	Mn (kDa)	**Mw (kDa)**	PDI[1]
6300	6900	**8300**	1.20

[1]Polydispersity index of the polymer with a minimum value of 1 (= pure monodisperse sample).

Estimation of the deacetylation degree (DD)

From the spectrum recorded in D_2O, the massif from 3.98 to 3.32 ppm corresponds to H3-4-5-6 of dCS. It is used as reference peak. Integration of the singlet at 1.97 ppm leads to 0.63. This singlet corresponds to the 3 H of the acetyl groups from the acetylglucosamine units of chitosan.

$$DD = (1 - AD) \times 100 \text{ where } AD = \frac{\int \delta\ (1.97\ \text{ppm})}{nb\ (H7)} = \frac{0.63}{3} = 0.21\text{, hence DD} = 79\%$$

Estimation of the molar mass of one average dCS unit (M(dCS))

M(dCS) = M(glucosamine) x DD + M(acetylglucosamine) x AD

With a DD of 79%, M(dCS) = 161.16 x 0.79 + 203.20 x 0.21 = 170.0 g/mol

Estimation of the number of units per dCS chain (nb(dCS units))

$$\text{nb(dCS units)} = \frac{Mw(dCS)}{M(dCS)} = \frac{8300}{170.0} \approx 49 \text{ units}$$

<u>Characterization of dCS-Suc</u>

Prepared from dCS (DD = 79%)

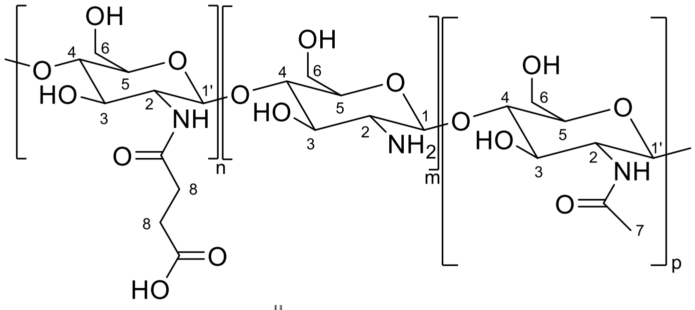

^1H NMR (600 MHz, D_2O/CD_3COOD 1/1): δ 5.20 (br, 0.05H, H1'), 4.94 (br, 0.16H, H1), 4.59 (br, 0.79H, H1'), 4.04 – 3.47 (m, 5H, H3-4-5-6), 3.21 (br, 1H, H2), 2.66 (br, 4H, H8).

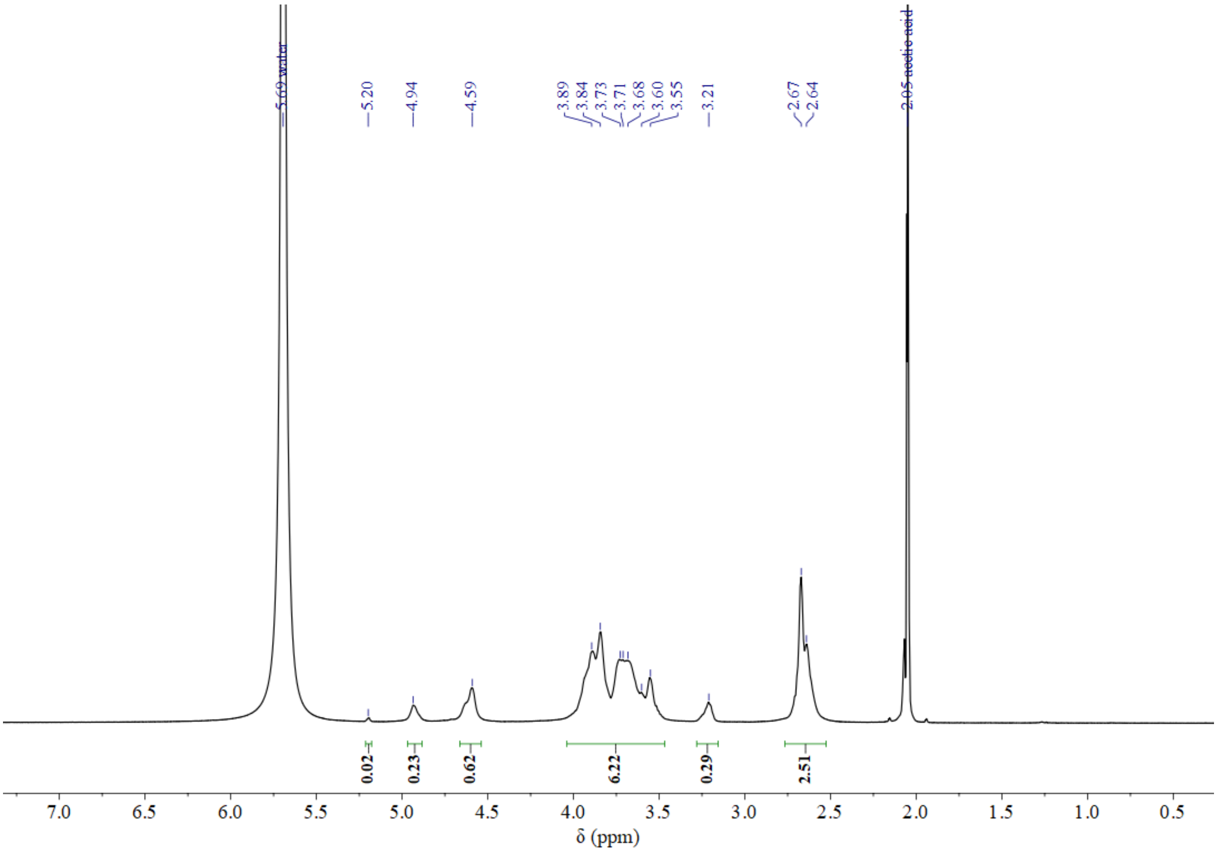

^1H NMR (400 MHz, D_2O): δ 5.19 (br, 0.05H, H1'), 4.88 (br, 0.16H, H1), 4.58 (br, 0.79H, H1'), 3.98 – 3.45 (m, 5H, H3-4-5-6), 3.16 (br, 1H, H2), 2.58 (br, 4H, H8), 2.06 (br, 3H, H7).

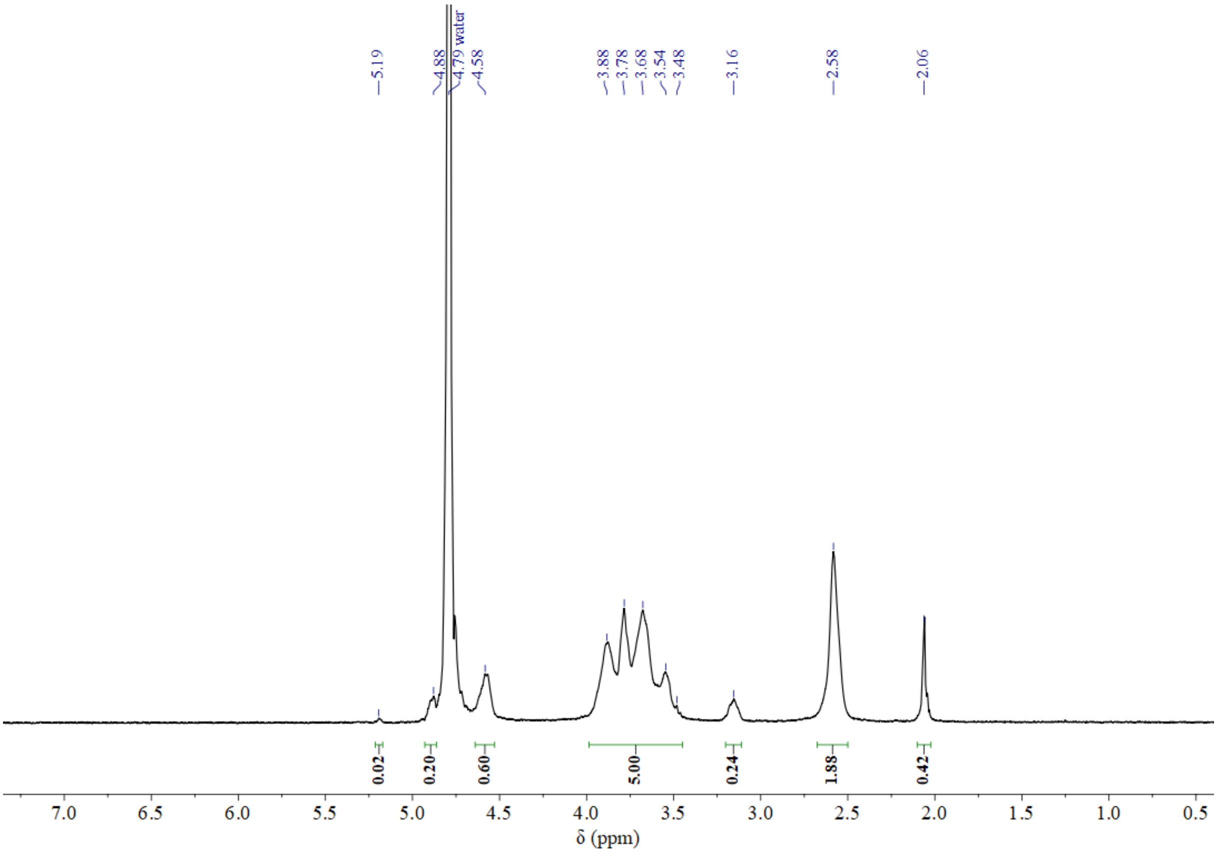

2D DOSY NMR (600 MHz, D_2O/CD_3COOD 1/1):

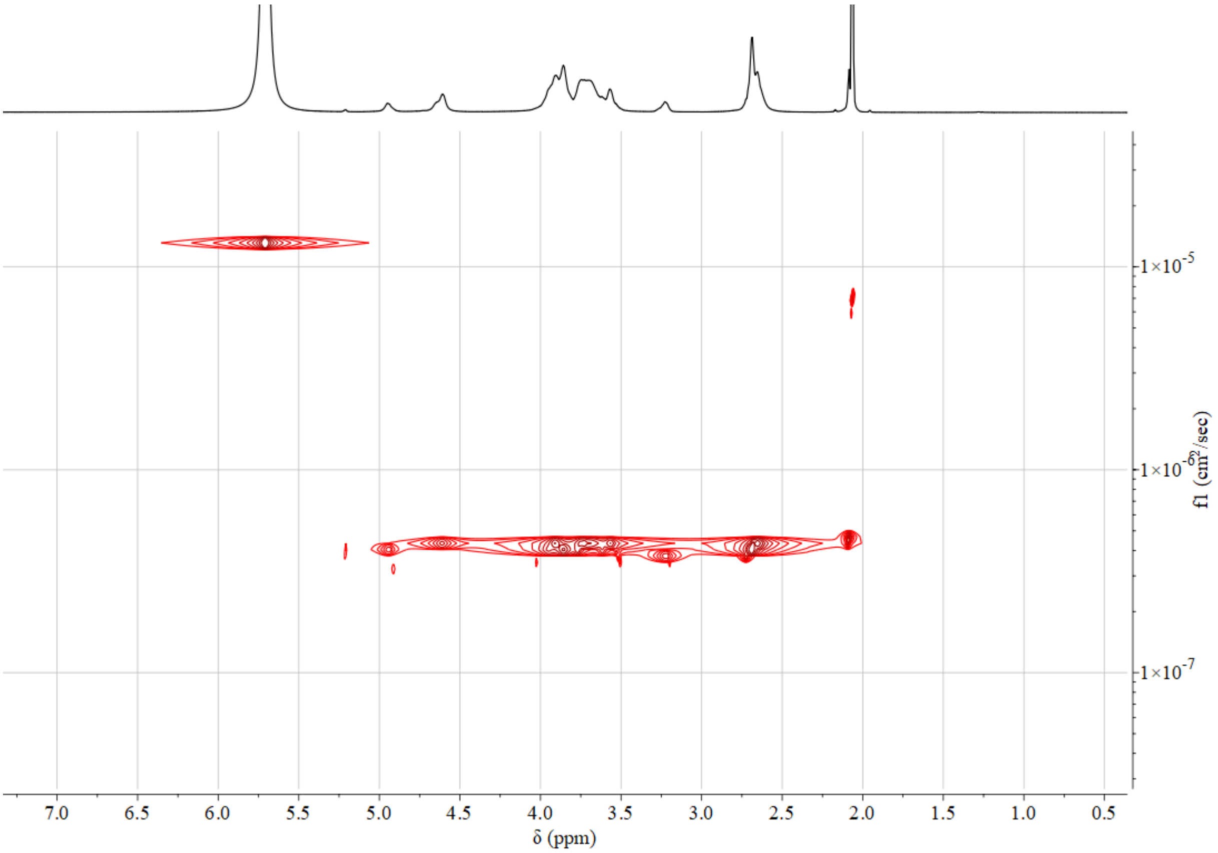

IR (cm^{-1}): 3270, 2930, 1645, 1549, 1378, 1154, 1064, 1029, 898, 622.

Calculation of the number of mol of reactive units (e.g glucosamine units) of dCS (n (glucosamine))

m(dCS) = m(glucosamine) + m(acetylglucosamine)

= M(glucosamine) x n(glucosamine) + M(acetylglucosamine) x n(acetylglucosamine)

We know that n(acetylglucosamine) = $\frac{AD}{DD}$ x n(glucosamine)

Hence: n(glucosamine) = $\frac{m(dCS)}{M(glucosamine)+(AD/DD)\ x\ M(acetylglucosamine)}$

Here: n(glucosamine) = $\frac{1.00}{161.16 + 0.25\ x\ 203.20}$ = 4.72 mmol

Estimation of grafting degree of succinyl group on dCS (GD_{Suc})

Integrations for H1 + H1′ equal 1. From the spectrum recorded in D_2O/acetic acid-d^4 (1/1), the integration for the succinyl group (2.66 ppm) leads to 2.51 H. Each succinyl group accounts for 4 H.

GD_{Suc} = $\frac{2.51}{4}$ = 0.628 or 63%.

Estimation of the molar mass of one average dCS-Suc unit (M(dCS-Suc))

M(dCS-Suc) = GD_{Suc} x M(dCS-Suc unit) + AD x M(acetylglucosamine) + (1 - GD_{Suc} - AD) x M(glucosamine)

= 0.63 x 261.16 + 0.21 x 203.20 + (1.00-0.63-0.21) x 161.16 = 233.0 g/mol

Estimation of the molecular weight of one dCS-Suc chain (MW(dCS-Suc))

MW(dCS-Suc) = Mw(dCS) + GD_{Suc} x M_{Suc} x nb(dCS units)

= 8300 + 0.63 x 101.08 x 49 = 11.4 kDa

with 49 being the number of units per dCS chain (see procedure of CS depolymerization for details).

Characterization of dCS-Suc-LPEI

Prepared from dCS-Suc (GD_{Suc} = 63%, DD = 79%)

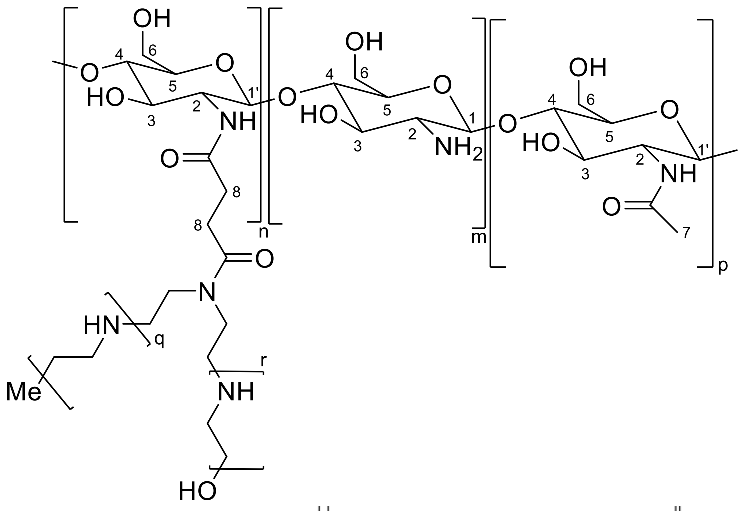

^{1}H NMR (600 MHz, D_2O/CD_3COOD 1:1): δ 4.93 (br, 0.16H, H1), 4.60 (br, 0.84H, H1'), 4.14 – 2.96 (m, 39H, H2-3-4-5-6 + H of LPEI), 2.66 (br, 4H, H8).

δ 2.79, 2.47, 1.25, 1.10 are impurities from the starting LPEI (present in all commercial batches).

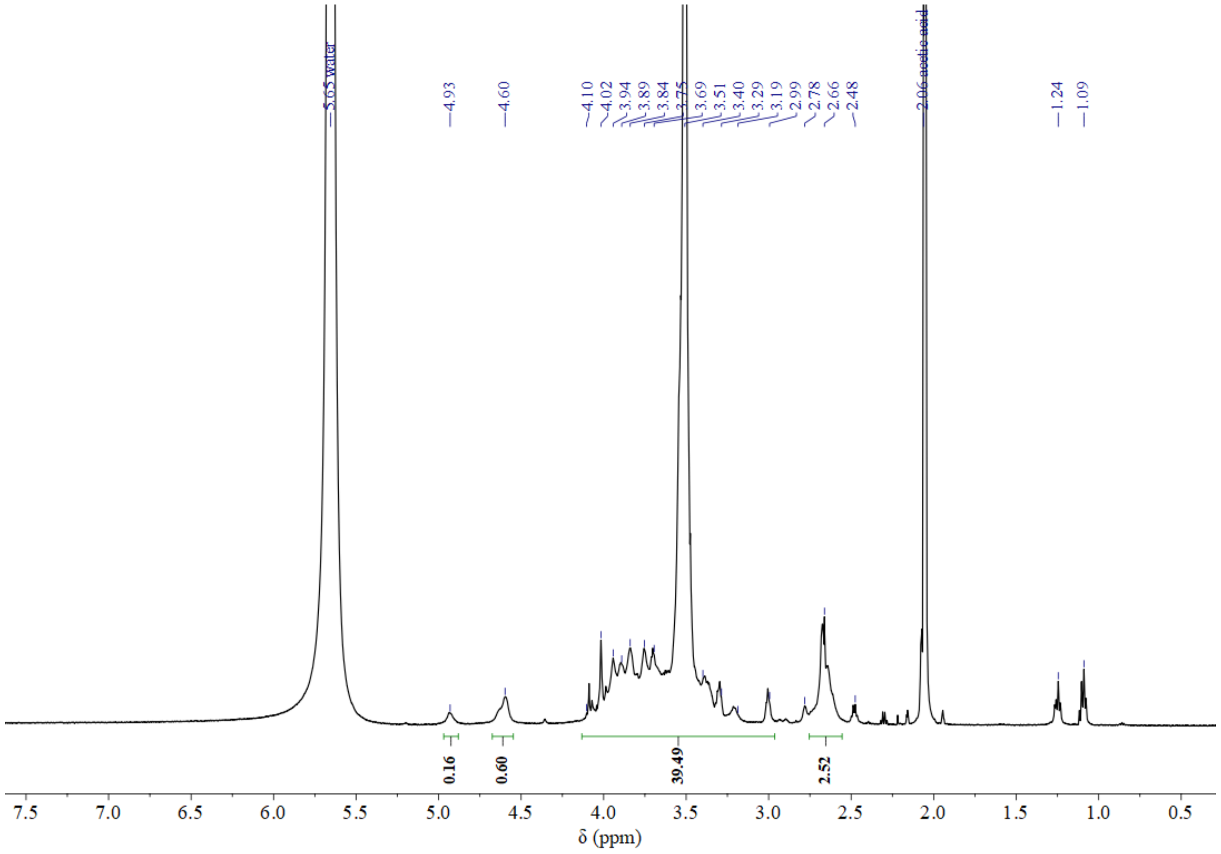

^{1}H NMR (400 MHz, D_2O): δ 4.59 (br, 0.84H, H1'), 4.48 (br, 0.16H, H1) 4.12 – 2.64 (m, 39H, H2-3-4-5-6 + H of LPEI), 2.63-2.44 (br, 4H, H8), 2.06 (s, 3H, H7). δ 1.21, 1.10 are impurities from the starting LPEI (present in all commercial batches).

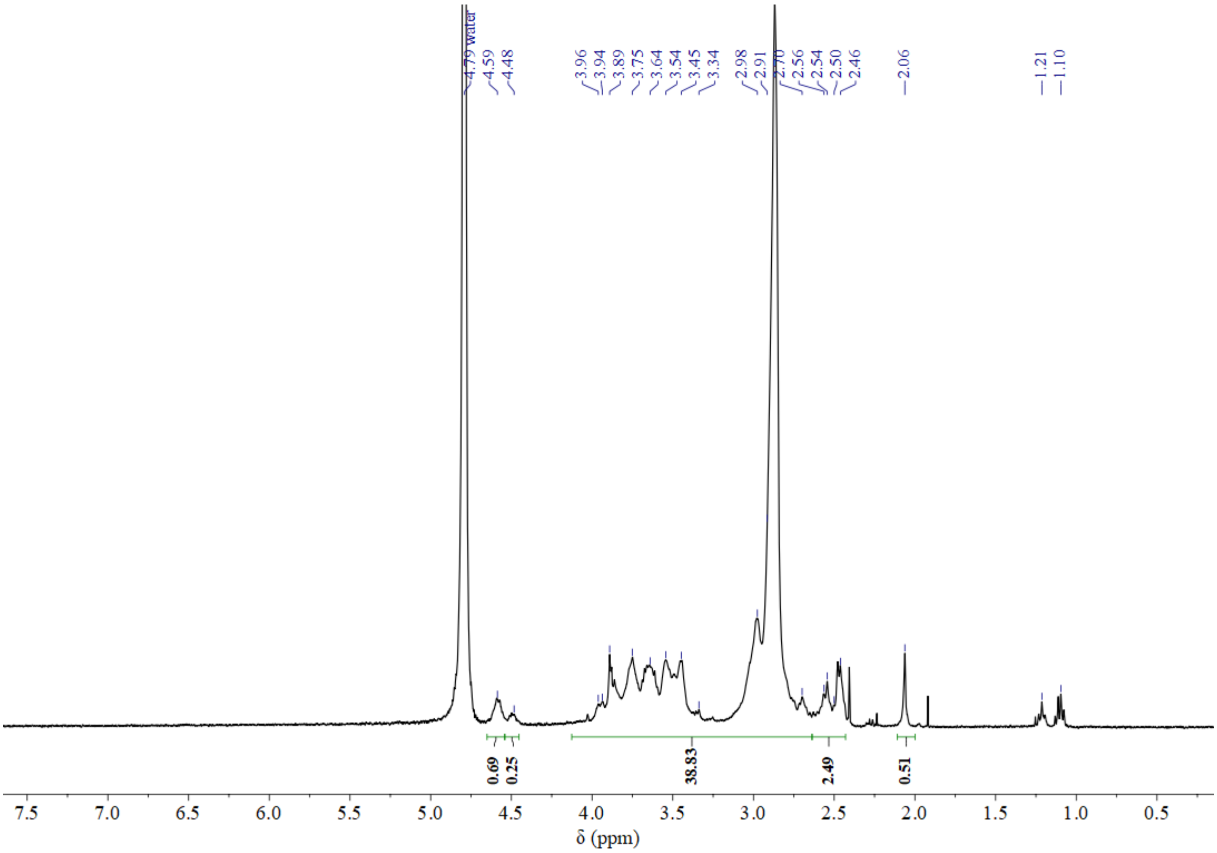

2D DOSY NMR (600 MHz, D_2O/CD_3COOD 1:1):

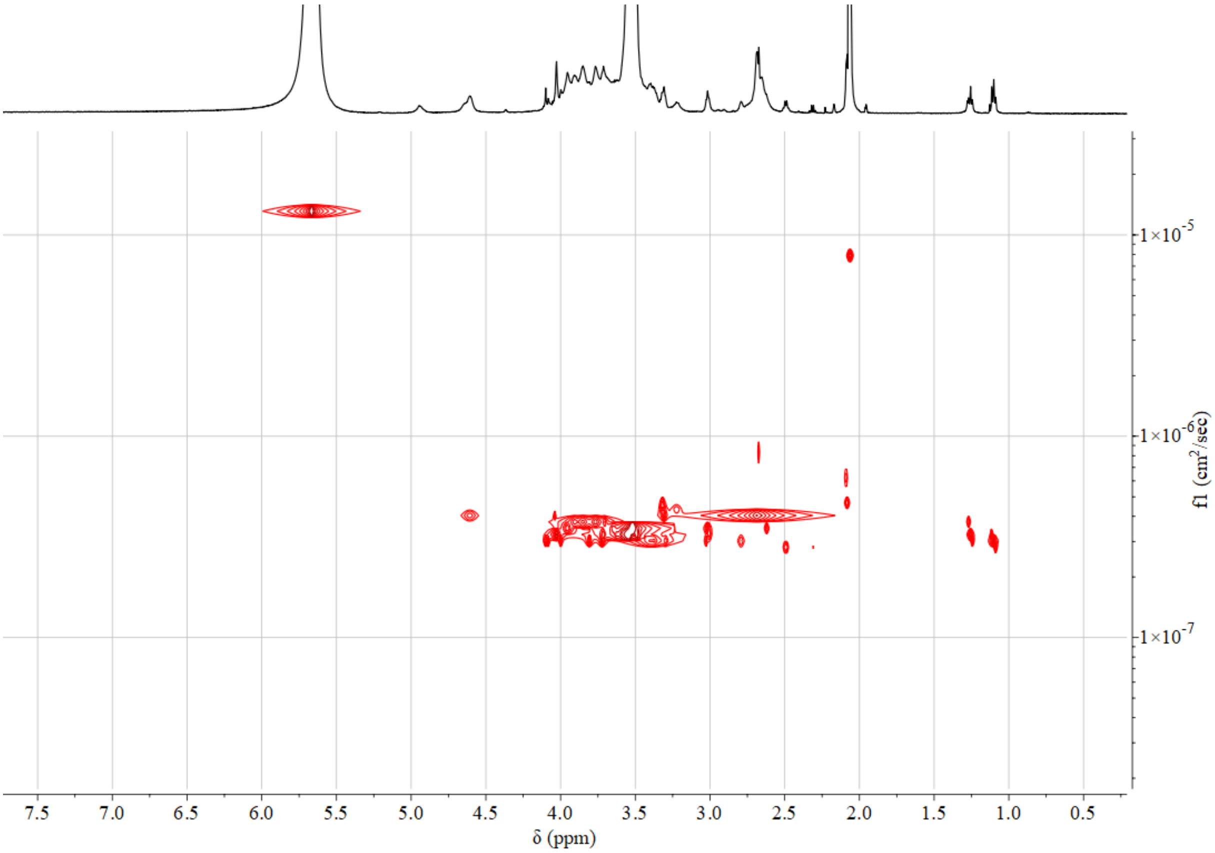

IR (cm^{-1}): 3272, 2917, 2847, 1651, 1555, 1464, 1408, 1372, 1303, 1109, 1063, 1030, 899, 812, 646.

Calculation of the number of mol of reactive units (e.g succinylated glucosamine units) of dCS-Suc (n(reactive units)

As GD_{Suc} = 63% and DD = 79%, the molar mass of one average unit of dCS-Suc is the following:

M(dCS-Suc) = GD_{Suc} x M(dCS-Suc unit) + AD x M(acetylglucosamine) + (1 - GD_{Suc} - AD) x M(glucosamine)

= 0.63 x 261.16 + 0.21 x 203.20 + (1-0.63-0.21) x 161.16 = 233.0 g/mol

Hence, the average number of mol of dCS-Suc is:

$$\text{average n} = \frac{m(dCS-Suc)}{M(dCS-Suc)} = \frac{0.200}{233.0} = 0.860 \text{ mmol}$$

With only 63% of the dCS units available for reaction with DMTMM and LPEI:

n(reactive units) = 0.860 x 0.63 = 0.542 mmol.

Estimation of grafting degree of LPEI on dCS-Suc (GD_{LPEI})

From the spectrum recorded in acetic acid-d^4/D_2O (1/1), the peak from the succinyl group (2.66 ppm) is used as the reference peak. From the previous step, the integration for this peak equals to 2.52 (GD_{Suc} = 63%). This value is reported on the present spectrum. The integration of the massif from 4.14 to 2.96 ppm leads to a total number of 39.5 H, representing H2-3-4-5-6 from dCS units and CH_2 of LPEI chain. The CH_2 (LPEI) account for 33.5 H. In one LPEI 2.5 kDa chain, there are 58 units of ethylenimine, accounting for 232 H.

$$\text{Hence, } GD_{LPEI} = \frac{33.5}{232} \text{ x } 100 = 14\%$$

For this calculation, we considered that each LPEI chain underwent a single grafting reaction to dCS-Suc. GD_{LPEI} was estimated for every dCS units, including the acetylated (and not reactive) ones.

Estimation of the molecular weight of one dCS-Suc-LPEI chain (MW(dCS-Suc-LPEI))

MW(dCS-Suc-LPEI) = Mw(dCS) + GD_{Suc} x M_{Suc} x nb(dCS units) + GD_{LPEI} x MW(LPEI) x nb(dCS units)

= 8300 + 0.63 x 101.08 x 49 + 0.14 x 2500 x 49 = 28.6 kDa

with 49 being the number of units per dCS chain (see procedure of CS depolymerization for details).

Characterization of dCS-Suc-BPEI
from dCS-Suc (DD = 83%, GD_{Suc} = 45%) and BPEI 1.8 kD

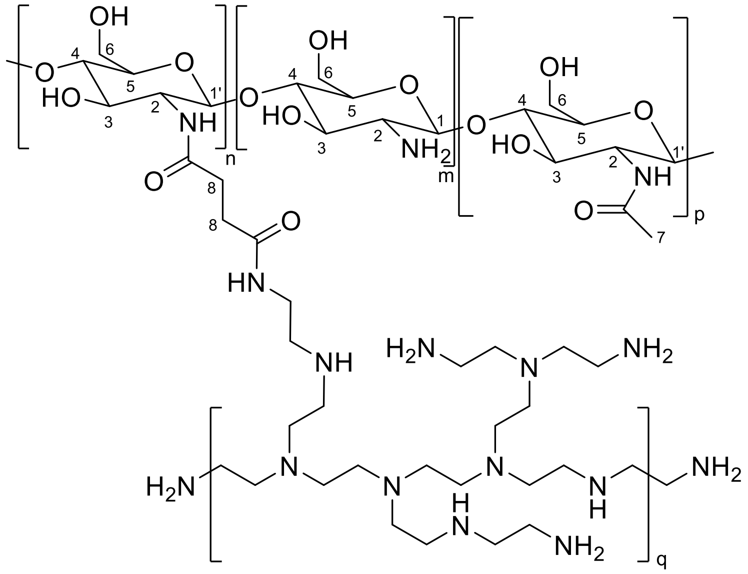

^{1}H NMR (400 MHz, D_2O/CD_3COOD 1:1): δ 5.25 (br, 0.05H, H1’), 4.94 (br, 0.38H, H1), 4.60 (br, 0.57H, H1’), 4.13 – 2.75 (m, 36H, H2-3-4-5-6 + H of BPEI), 2.69 (br, 4H, H8).

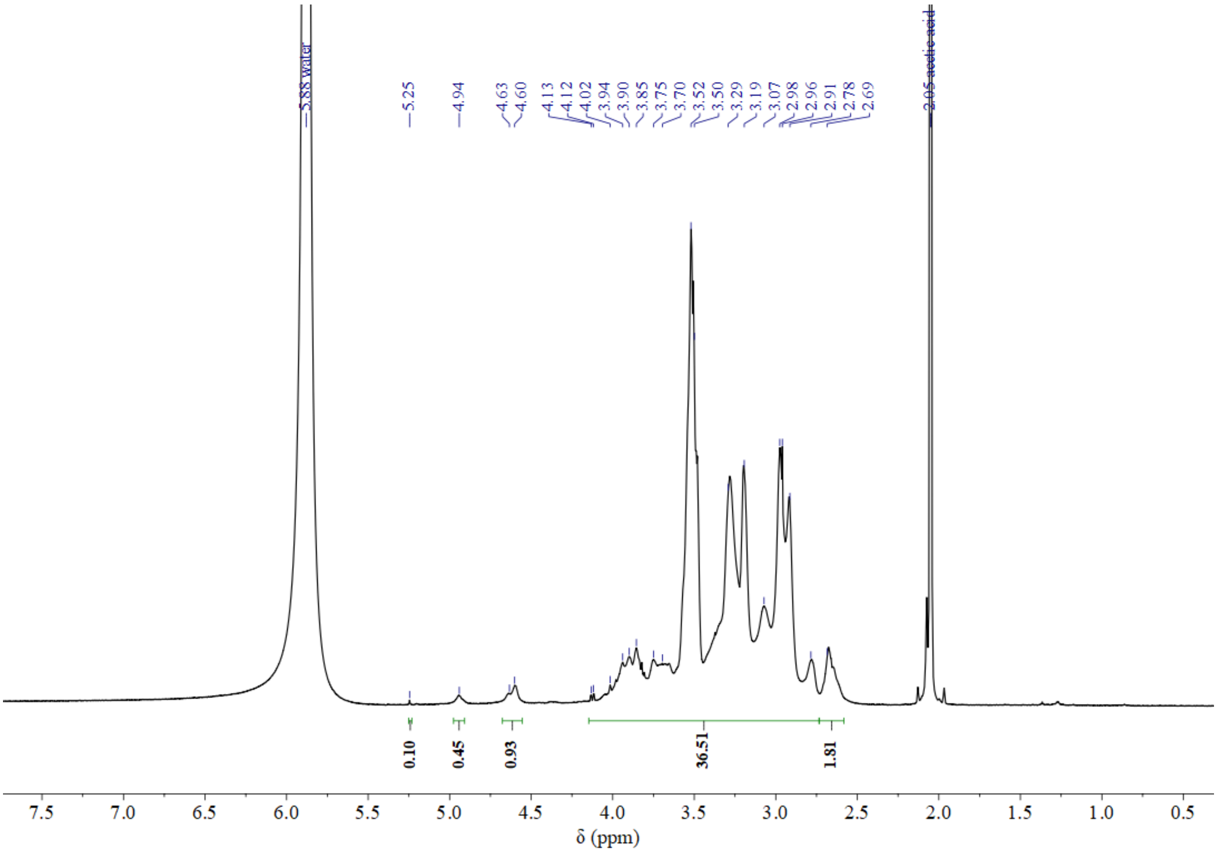

^{1}H NMR (400 MHz, D_2O): δ 4.58 (br, H, H1’), 4.01 – 3.08 (m, 6H, H2-3-4-5-6), 3.07 – 2.47 (br, 30H, H8 + H of BPEI), 2.06 (s, 3H, H7).

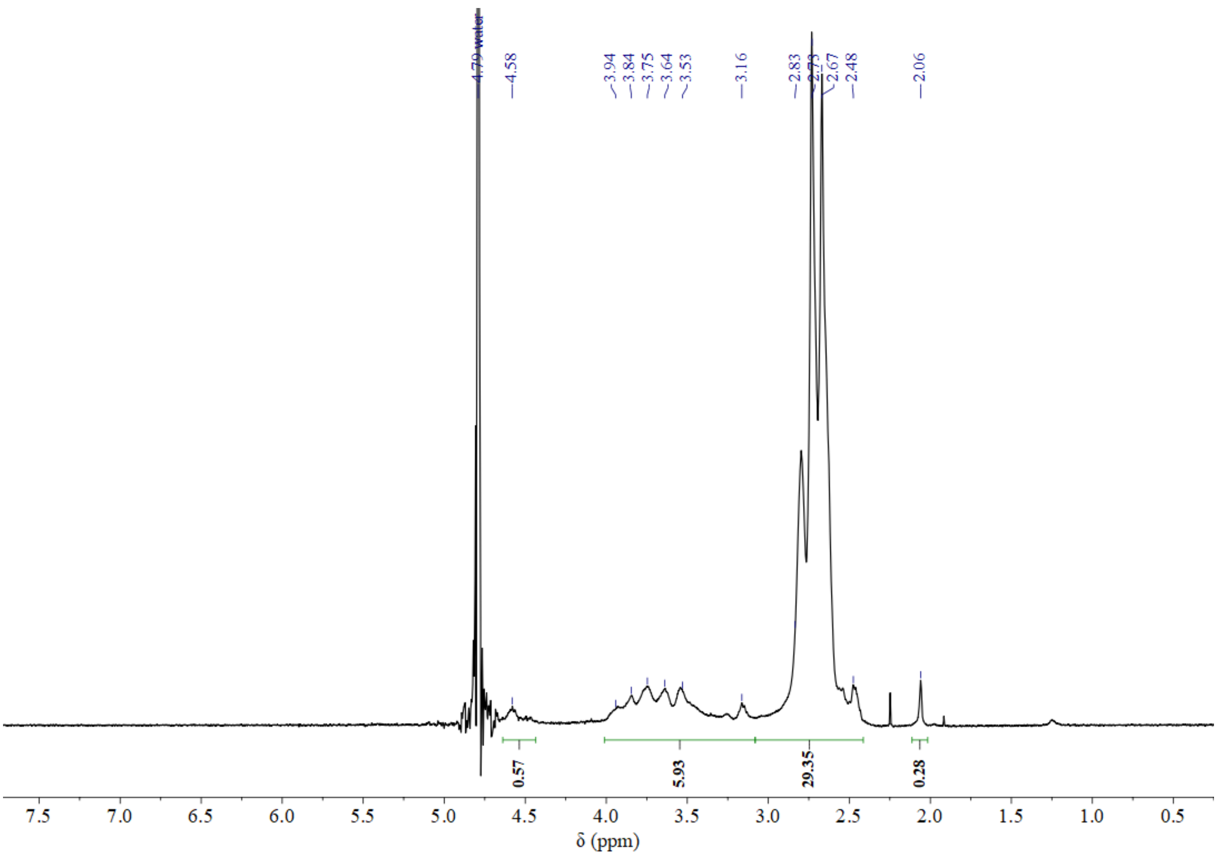

2D DOSY NMR (800 MHz, D_2O/CD_3COOD 1:1):

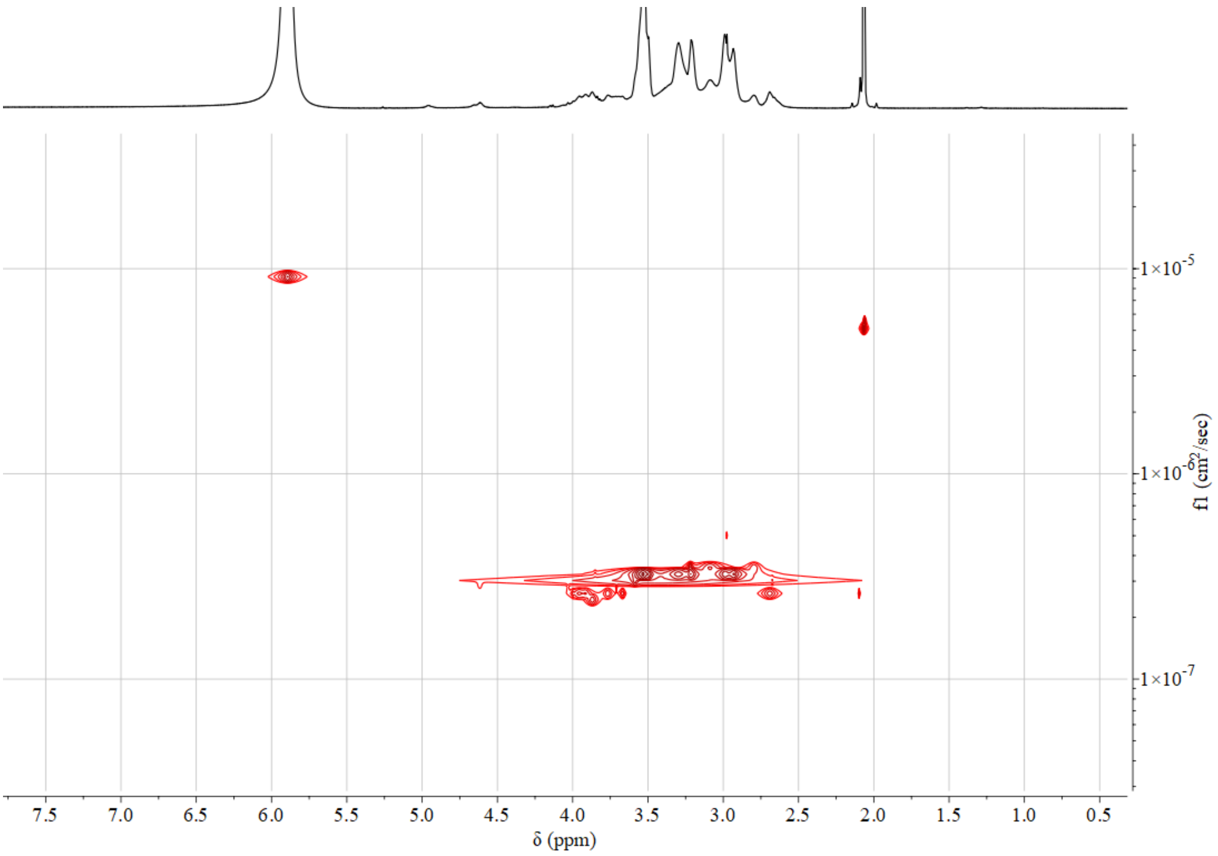

Calculation of the number of mol of reactive units (e.g succinylated glucosamine units) of dCS-Suc (n(dCS-Suc average))

n(dCS-Suc average) = $\frac{m(dCS-Suc)}{M(dCS-Suc)} = \frac{0.200}{194.3}$ = 1.03 mmol

Calculation of the mass of BPEI (m(BPEI)) needed depending on the reactive groups of BPEI

BPEI is composed of primary (I), secondary (II) and tertiary (III) amines. Due to steric hindrance, we considered that only primary amines would react. From the supplier data, for BPEI 1.2 kDa (50 wt% in water): I/II/III amines ratio = 1/0.6/0.9 meaning that only 40% of amino groups of BPEI are considered as reactive functionalities. The same ratio was considered for BPEI 1.8 kDa.

n(BPEI chains) = $\frac{n(reactive\ BPEI\ groups)}{ratio(I\ amines\ BPEI)} = \frac{0.638}{0.4}$ = 1.60 mmol

m(BPEI) = n(BPEI chains) x MW(BPEI) = 1.60 x 1800 = 2.87 g

Estimation of grafting degree of BPEI on dCS-Suc (GD_{BPEI})

From the spectrum recorded in acetic acid-d^4/D_2O (1/1), the peak from the succinyl group (2.69 ppm) is used as the reference peak. From the previous step, the integration for this peak equals to 1.81 (GD_{Suc} = 45%). This value is reported on the present spectrum. The integration of the massif from 4.13 to 2.75 ppm leads to a total number of 36.5 H, representing H2-3-4-5-6 from dCS units and CH_2 of BPEI chain. The CH2 (BPEI) account for 30.5 H. In one BPEI 1.8 kDa chain, there are 42 units of ethylenimine accounting for 168 H.

Hence, $GD_{BPEI} = \frac{30.5}{168}$ x 100 = 18%

For this calculation, we considered that each BPEI chain underwent a single grafting reaction to dCS-Suc. GD_{BPEI} was estimated for every dCS units, including the acetylated (and not reactive) ones.

Estimation of the molar mass of one average dCS-Suc-BPEI unit (M(dCS-Suc-BPEI))

M(dCS-Suc-BPEI) = GD_{Suc} x M(dCS-Suc unit) + AD x M(acetylglucosamine) + (1 - GD_{Suc} - AD) x M(glucosamine) + GD_{BPEI} x MW(BPEI)

= 0.45 x 261.16 + 0.17 x 203.20 + (1.00-0.45-0.17) x 161.16 + 0.18 x 1800 = 576.0 g/mol

Estimation of the molecular weight of one dCS-Suc-BPEI chain (MW(dCS-Suc-BPEI))

MW(dCS-Suc-BPEI) = Mw(dCS) + GD_{Suc} x M_{Suc} x nb(dCS units) + GD_{BPEI} x MW(BPEI) x nb(dCS units)

= 7800 + 0.45 x 101.08 x 46 + 0.18 x 1800 x 46 = 24.8 kDa

with 46 being the number of units per dCS chain (see procedure of depolymerization of CS for details).

Characterizations of dCS-NSucBPEI-OPEG-SH

Prepared from dCS-Suc-BPEI (GD_{Suc} = 47%, GD_{BPEI}= 11%, DD = 80%) and HS-PEG-NH_2.HCl 2 kDa

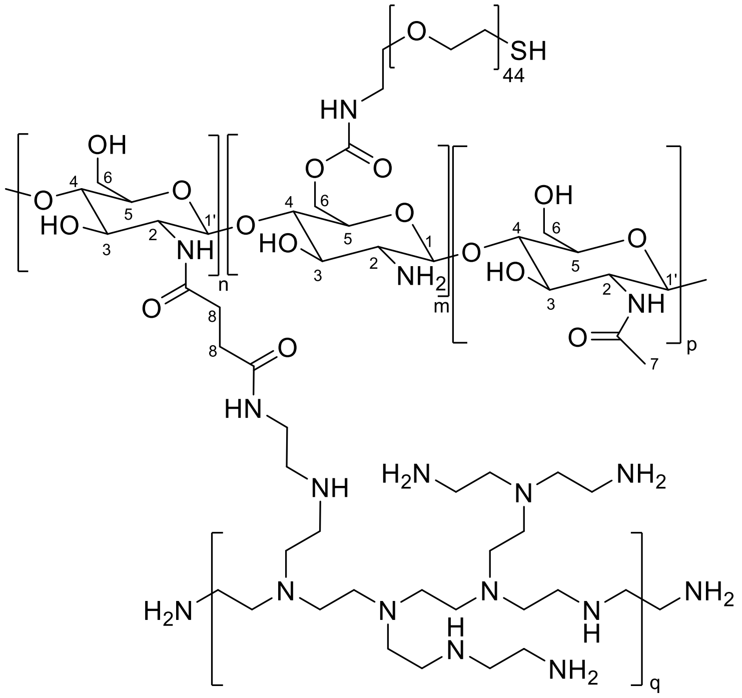

^{1}H NMR (600 MHz, D_2O/CD_3COOD 1:1): δ 4.94 (br, 0.33H, H1), 4.60 (br, 0.67H, H1′), 4.02 – 2.74 (m, 70H, H2-3-4-5-6 + H of BPEI + H of PEG), 2.66 (br, 4H, H8).

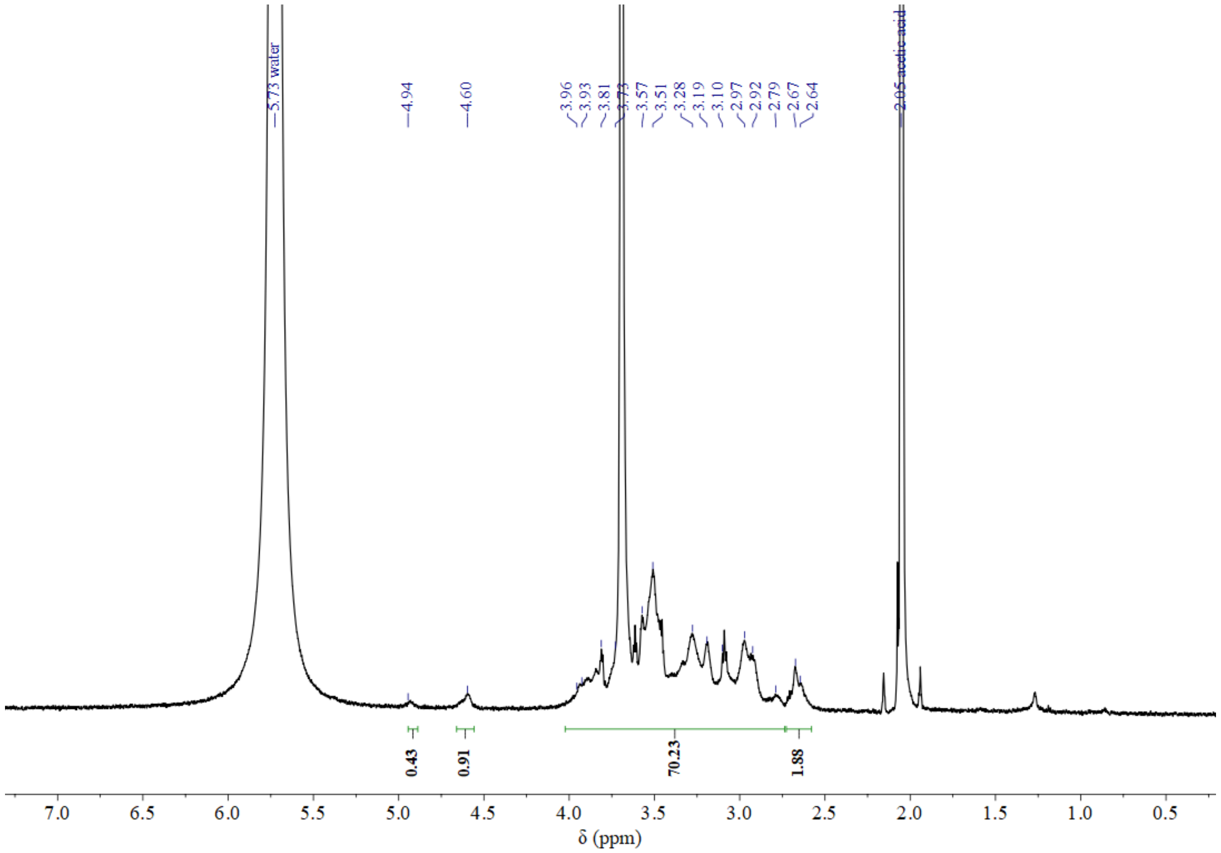

^{1}H NMR (400 MHz, D_2O): δ 4.65 – 4.43 (br, 1H, H1 + H1′), 4.00 – 2.41 (m, 66H, H2-3-4-5-6 + H of BPEI + H of PEG + H8), 2.06 (s, 3H, H7).

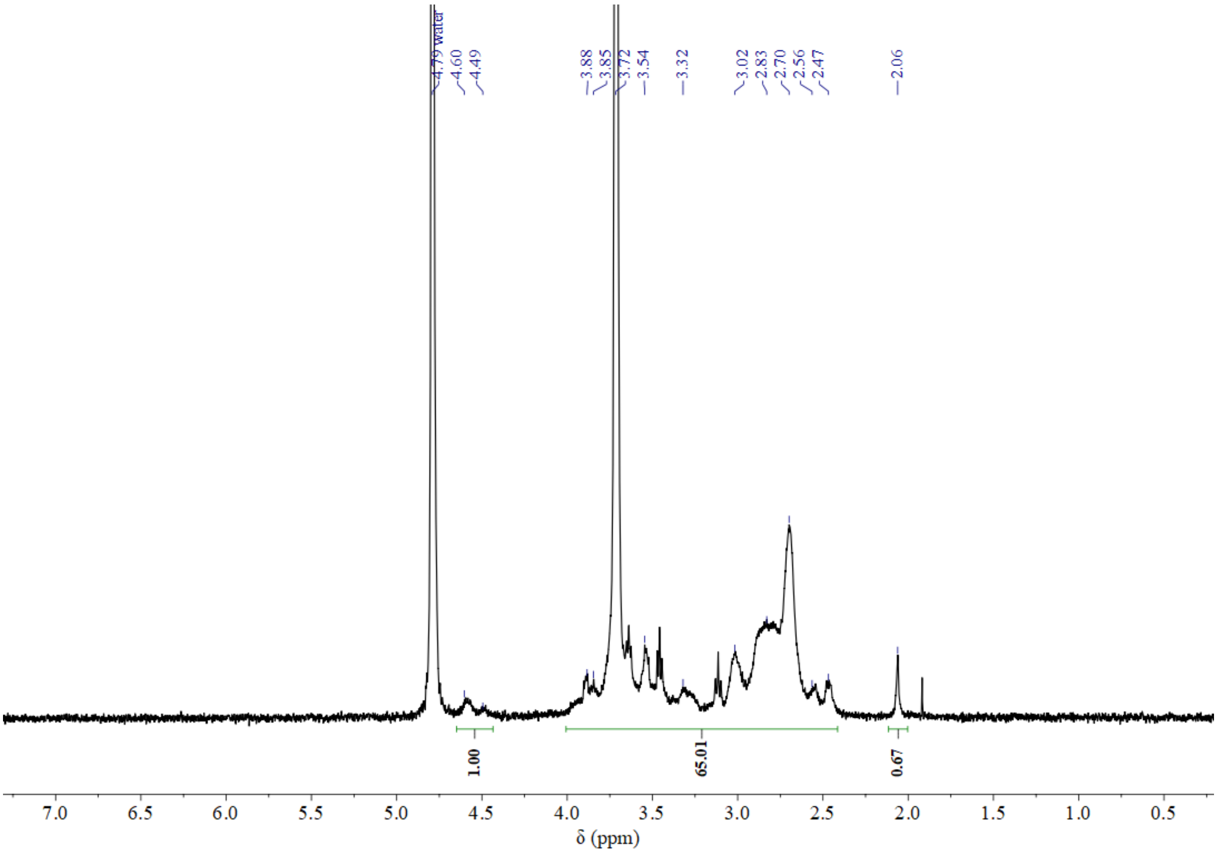

2D DOSY NMR (600 MHz, D_2O/CD_3COOD 1:1):

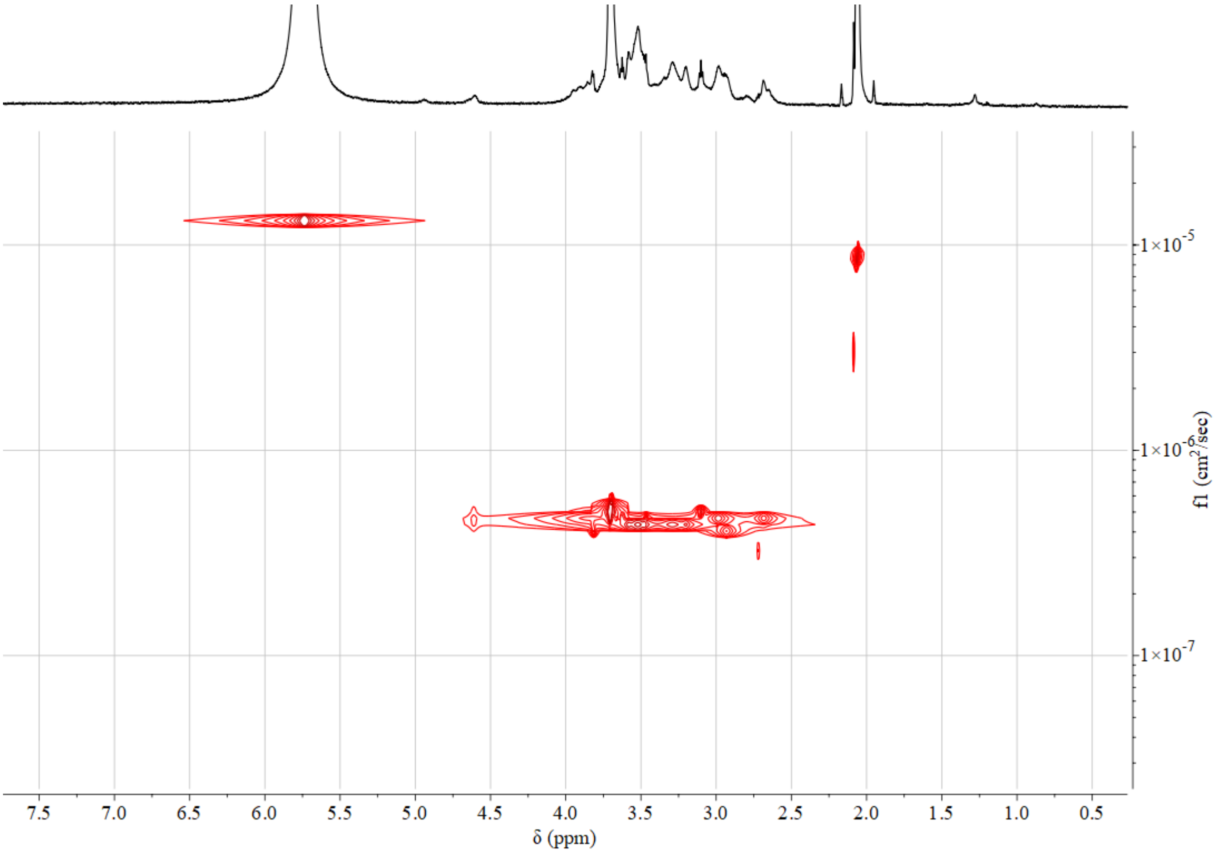

Estimation of the grafting degree of PEG (2 kDa) on dCS-Suc-BPEI (GD_{PEG})

From the spectrum recorded in D_2O/CD_3COOD (1/1), the peak from the succinyl group (2.66 ppm) is used as the reference peak. From the previous step, the integration for this peak equals to 1.88 (GD_{Suc} = 47%). The integration of the massif from 4.02 to 2.74 ppm leads to a total number of 70 H, representing H2-3-4-5-6 from dCS units (6 H), CH_2 of PEG chain and CH_2 of BPEI chain (GD_{BPEI} = 11%). The CH_2 (PEG) account for 46 H. In one PEG 2 kDa chain, there are 180 H.

Hence, $GD_{PEG} = \frac{46}{180} \times 100 = 26\%$

Estimation of the molecular weight of one dCS-NSucBPEI-OPEG-SH chain (MW(dCS-NSucBPEI-OPEG-SH))

MW(dCS-NSucBPEI-OPEG-SH) = Mw(dCS) + GD_{Suc} x M_{Suc} x nb(dCS units) + GD_{BPEI} x MW(BPEI) x nb(dCS units) + GD_{PEG} x MW(PEG) x nb(dCS units)

= 7800 + 0.47 x 101.08 x 46 + 0.11 x 1800 x 46 + 0.26 x 2042 x 46

= 43.5 kDa

with 46 being the number of units per dCS chain (see procedure of depolymerization of CS for details).

Characterizations of dCS-NSuc-OPEG-SH

from dCS-Suc (GD_{Suc} = 44%, DD = 85%) and HS-PEG-NH_2.HCl 2 kDa

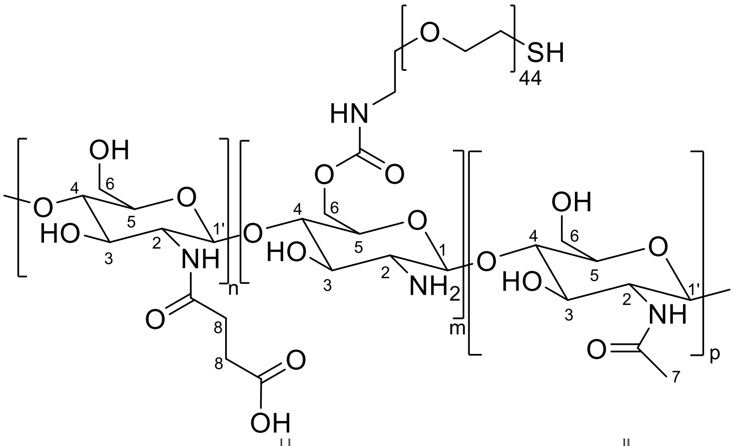

^{1}H NMR (400 MHz, D_2O/CD_3COOD 1:1): δ 4.93 (br, 0.41H, H1), 4.60 (br, 0.59H, H1'), 4.18 – 3.03 (m, 30H, H2-3-4-5-6 + H of BPEI), 2.94 (t, 2H, -C$H_2$$CH_2$SH), 2.66 (br, 4H, H8).

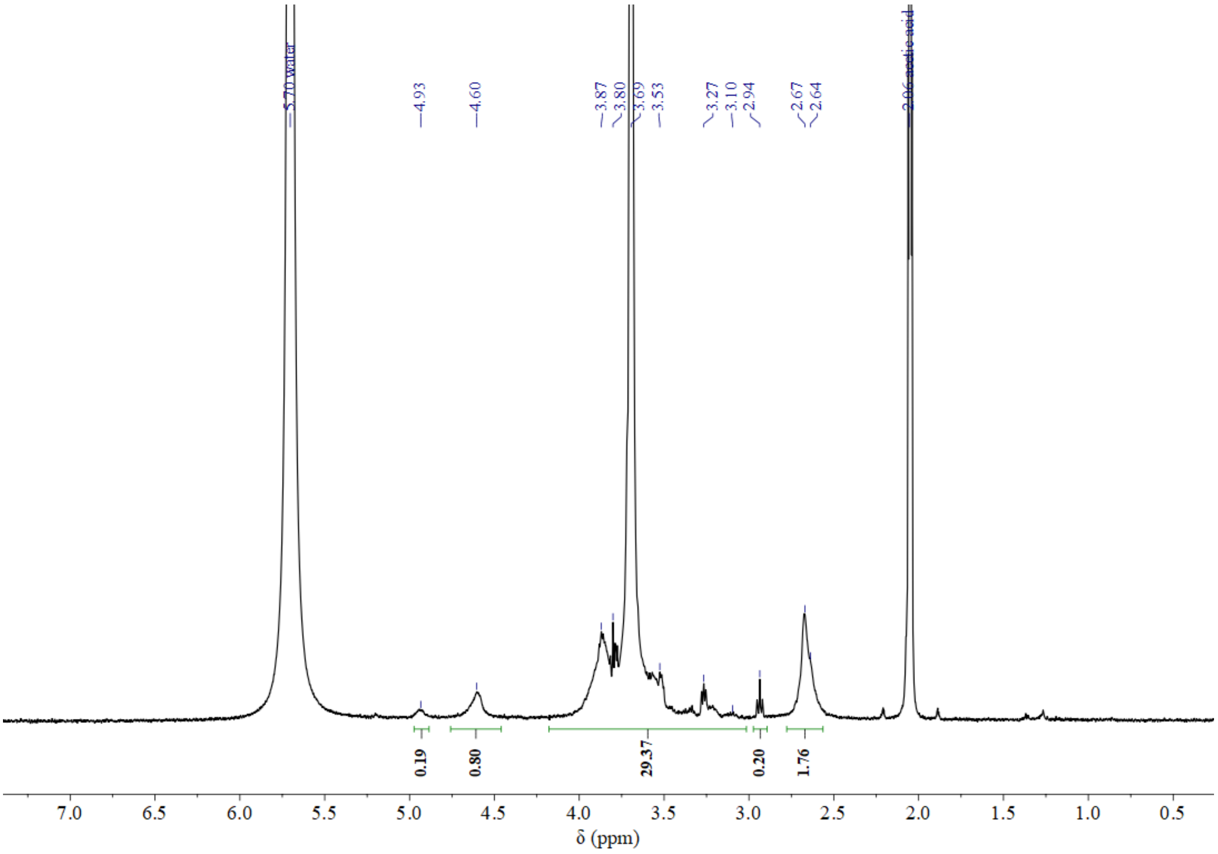

2D DOSY NMR (400 MHz, D_2O/CD_3COOD 1:1):

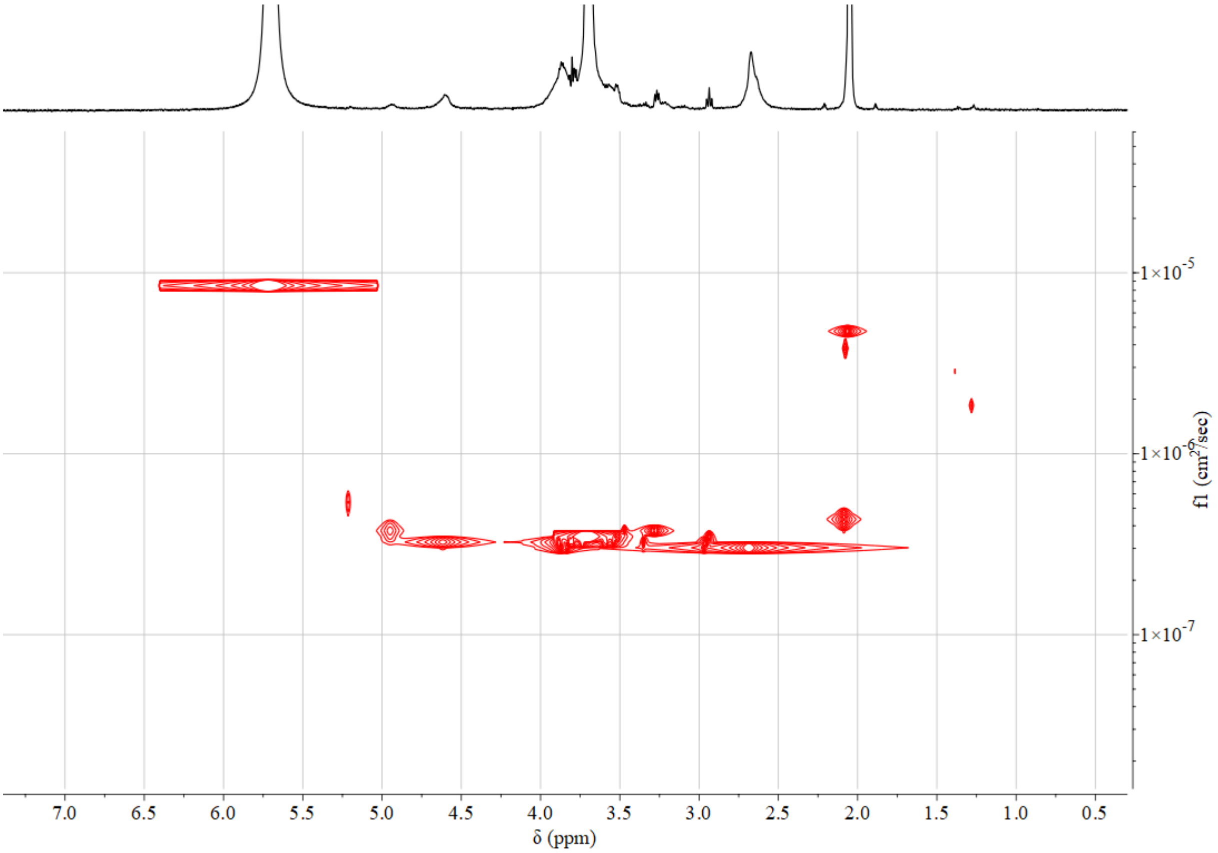

Estimation of the grafting degree of PEG (2 kDa) on dCS-Suc (GD_{PEG})

From the spectrum recorded in D_2O/CD_3COOD (1/1), the peak from the succinyl group (2.66 ppm) is used as the reference peak. From the previous step, the integration for this peak equals to 1.76 (GD_{Suc} = 44%). The integration of the massif from 4.18 to 3.03 ppm and of the triplet at 2.94 ppm leads to a total number of 29.6 H, representing H2-3-4-5-6 from dCS units, CH_2 of PEG main chain and -$\mathit{CH_2}CH_2SH$. The CH_2 (PEG) account for 29.6 H. In one PEG 2 kDa chain, there are 180 H.

Hence, $GD_{PEG} = \frac{23.6}{180} \times 100 = 14\%$

Estimation of the molar mass of one average dCS-NSuc-OPEG-SH unit (M(dCS-NSuc-OPEG-SH))

M(dCS-NSuc-OPEG-SH) = GD_{Suc} x M(dCS-Suc unit) + AD x M(acetylglucosamine) + (1 - GD_{Suc} - AD) x M(glucosamine) + GD_{PEG} x MW(PEG)

= 0.44 x 261.16 + 0.15 x 203.20 + (1.00-0.44-0.15) x 161.16 + 0.14 x 2042

= 498.6 g/mol

Estimation of the molecular weight of one dCS-NSuc-OPEG-SH chain (MW(dCS-NSuc-OPEG-SH))

MW(dCS-NSuc-OPEG-SH) = Mw(dCS) + GD_{Suc} x M_{Suc} x nb(dCS units) + GD_{PEG} x MW(PEG) x nb(dCS units)

= 7800 + 0.44 x 101.08 x 46 + 0.14 x 2042 x 46

= 23.0 kDa

with 46 being the number of units per dCS chain (see procedure of depolymerization of CS for details).

Characterizations of dCS-NSucLPEI-OPEG-SH

from dCS-NSuc-OPEG-SH (GD_{Suc} = 44%, GD_{PEG} = 14%, DD = 85%) and LPEI 2.5 kDa

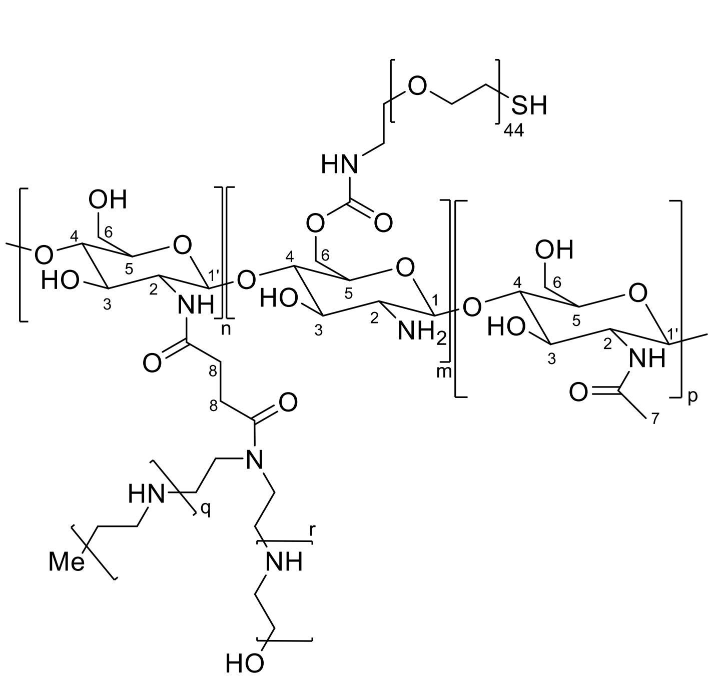

^{1}H NMR (600 MHz, D_2O/CD_3COOD 1:1): δ 4.95 (br, 0.41H, H1), 4.61 (br, 0.59H, H1′), 4.12 – 3.20 (m, 82H, H2-3-4-5-6 + H of PEG + H of LPEI), 2.67 (br, 4H, H8).

δ 2.80, 2.49, 1.26, 1.10 are impurities from the starting LPEI.

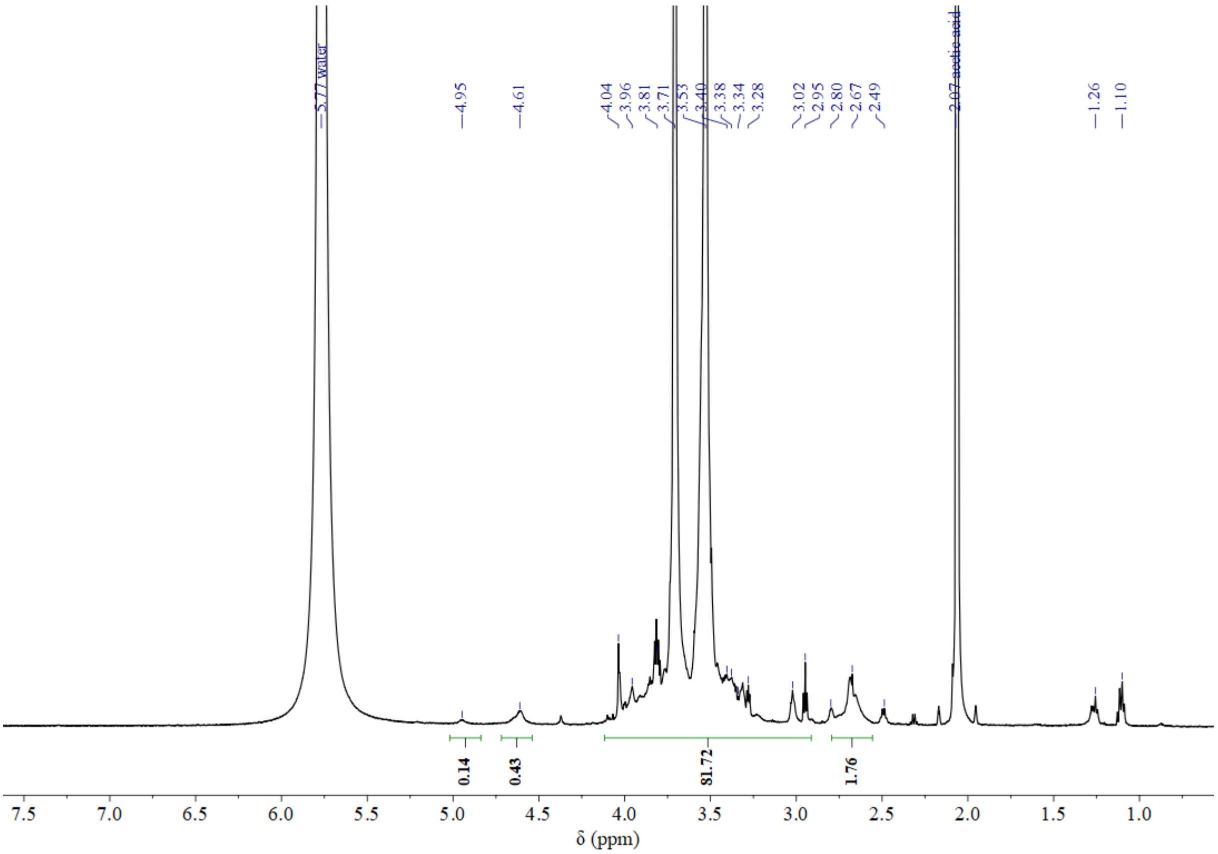

2D DOSY NMR (600 MHz, D_2O/CD_3COOD 1:1):

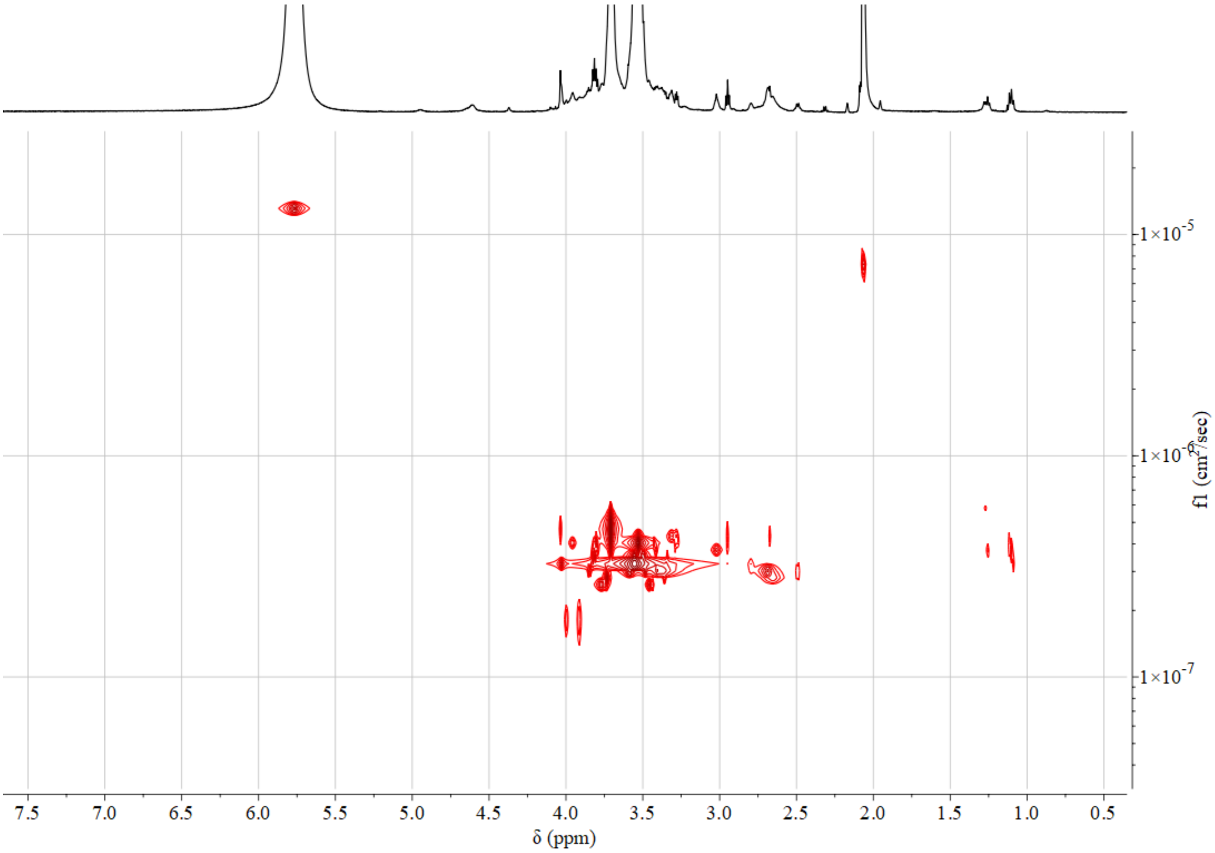

Calculation of the number of mol of reactive units (e.g succinylated glucosamine units) of dCS-NSuc-OPEG-SH (average n)

As GD_{Suc} = 44% and DD = 85%, the molar mass of one average unit of dCS-Suc is the following: M(dCS-NSuc-OPEG-SH) = GD_{Suc} x M (dCS-Suc unit) + (1 - GD_{Suc} - AD) x M(glucosamine) + AD x M(acetylglucosamine) + GD_{PEG} x MW(PEG)

= 0.44 x 261.16 + (1-0.44-0.15) x 161.16 + 0.15 x 203.20 + 0.14 x 2042

= 498.6 g/mol

Average number of mol of dCS-Suc: average n = $\frac{m(dCS-NSuc-OPEG-SH)}{M(dCS-NSuc-OPEG-SH)} = \frac{0.050}{498.6}$ = 0.100 mmol.

Estimation of the grafting degree of LPEI (2.5 kDa) on dCS-NSuc-OPEG-SH (GD_{LPEI})

From the spectrum recorded in D_2O/CD_3COOD (1/1), the peak from the succinyl group (2.67 ppm) is used as the reference peak. From the previous step, the integration for this peak equals to 1.76 (GD_{Suc} = 44%). The integration of the massif from 4.12 to 3.20 ppm leads to a total number of 86.1 H, representing H2-3-4-5-6 from dCS units, CH_2 of PEG chain and CH_2 of LPEI chain. The CH_2 (LPEI) account for 52.1. In one LPEI 2.5 kDa chain, there are 232 H.

Hence, $GD_{LPEI} = \frac{52.1}{232}$ x 100 = 22%

Estimation of the molecular weight of one dCS-NSucBPEI-OPEG-SH chain (MW(dCS-NSucLPEI-OPEG-SH))

MW(dCS-NSucLPEI-OPEG-SH) = Mw(dCS) + GD_{Suc} x M_{Suc} x nb(dCS units) + GD_{LPEI} x MW(LPEI) x nb(dCS units) + GD_{PEG} x MW(PEG) x nb(dCS units)

= 7800 + 0.44 x 101.08 x 46 + 0.22 x 2500 x 46 + 0.14 x 2042 x 46

= 48.3 kDa

with 46 being the number of units per dCS chain (see procedure of depolymerization of CS for details).

Table S3: DNA exclusion assay to evaluate accessibility of DNA complexed with polymeric conjugates

Polymer	Colloidally stable complexes [c/p ratio]	DNA accessibility [%]
**Starting polymer**		
- *dCS*	32	-2 ± 2
- *LPEI*	32	6 ± 1
- *BPEI*	16	5 ± 3
**BPEI derivatives**		
- *dCS-Suc-BPEI-11*	2	0 ± 1
- *dCS-Suc-BPEI-13*	4	-8 ± 4
- *dCS-Suc-BPEI-67*	1	-5 ± 2
**PEG-conjugated BPEI derivatives**		
- *dCS-NSucBPEI-OPEG-SH*	2	2 ± 3
**LPEI derivatives**		
- *dCS-Suc-LPEI-11a*	1	-2 ± 4
- *dCS-Suc-LPEI-11b*	2	-5 ± 2
**PEG-conjugated LPEI derivatives**		
- *dCS-NSucLPEI-OPEG-SH*	2	-2 ± 3

Table S4: Scale up and reproducibility of dCS-Suc-LPEI synthesis

The scale up of dCS-Suc-LPEI synthesis led to the final compound **dCS-Suc-LPEI-14**.

Product	Total starting amount of precursor (g)	Final amount of desired product (g)	Yield (%)	GD (%)
dCS	24.6	4.40	18	-
dCS-Suc	4.0	3.65	67	63
dCS-Suc-LPEI-14	1.4	0.916	35	14

The reproducibility of dCS-Suc-LPEI synthesis was assessed during the scale up. The batches were first analyzed separately by ^1H and 2D-DOSY NMR before being mixed and analyzed again. 4 batches out of 7 were analyzed, leading to GD ranging from 12 to 14%. A 14% GD_{LPEI} was calculated from the analysis of the mixed batches. Overall, the GD were very similar considering that the starting polymer was characterized by a PDI of 1.2 and therefore not fully monodisperse. Additionally, estimation of GD via ^1H NMR necessarily leads to some variation depending on the operator and the way of integration. As a consequence, we tolerated a difference up to 5% for GD_{LPEI} between the different batches of the scale up, as well as between the different dCS-Suc-LPEI systems produced and described in this work, namely **dCS-Suc-LPEI-11a**, **dCS-Suc-LPEI-11b** and **dCS-Suc-LPEI-14**.

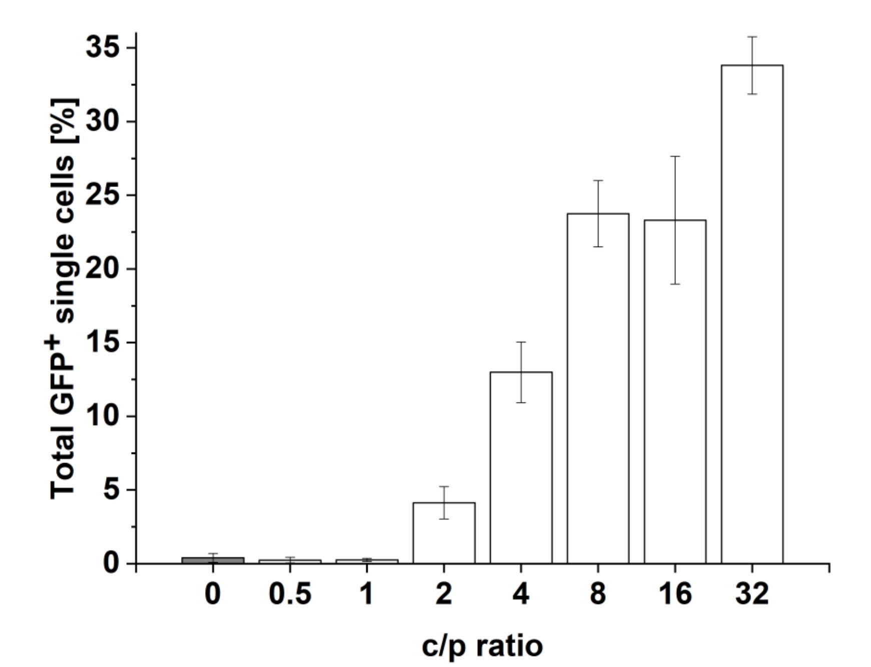

Figure S2: Quantitative analysis of *in vitro* GFP expression of the lead candidate **dCS-Suc-LPEI-14**. Flow cytometry experiments were conducted 48h after transfection, using the reporter gene nanovector-DNA (n.CAG.GFP1) and assessed based on total GFP positive cells as a function of c/p ratios. It should be noted that a c/p ratio of 2 offers the best compromise between transfection efficiency and cytotoxicity. Values are means ± SD, n = 3.
